# Bioscaffold materials resist infection and promote bone defect repair by regulating neutrophil function

**DOI:** 10.3389/fbioe.2025.1644625

**Published:** 2025-10-16

**Authors:** Jingyu Zhou, Shilang Xiong, Shiwei Liu, Zhigang Zhou, Min Liu, Hanrui Xi, Weihao Kong, Jianguo Zhou, Long Xiong

**Affiliations:** ^1^ Department of Orthopedics, The Second Affiliated Hospital, Jiangxi Medical College, Nanchang University, Nanchang, Jiangxi, China; ^2^ Institute of Orthopedics of Jiangxi Province, Nanchang, Jiangxi, China; ^3^ Jiangxi Provincial Key Laboratory of Spine and Spinal Cord Disease, Nanchang, Jiangxi, China; ^4^ Institute of Minimally Invasive Orthopedics, Nanchang University, Nanchang, Jiangxi, China; ^5^ Department of Orthopedics, Tenth People’s Hospital of Tongji University, Shanghai, China; ^6^ Department of Joint Surgery, Ganzhou People’s Hospital, Ganzhou, China

**Keywords:** neutrophils, scaffolds, bone tissue engineering, antibacterial, osteogenic

## Abstract

Infectious bone defects frequently encounter challenges related to bacterial infection and bone integrity. Neutrophils, being the initial responders to sites of inflammation, employ multiple mechanisms to eradicate bacteria, including phagocytosis, degranulation, the formation of neutrophil extracellular traps (NETs), and the oxidative respiratory burst. As a critical component of the human immune system, neutrophils play a pivotal role in modulating the inflammatory response, influencing the processes of osteogenesis and osteoclastogenesis, and impacting fracture healing. In the field of bone tissue engineering, the optimization of the chemical composition and morphology of scaffold materials can effectively modulate neutrophil behavior, thereby enhancing the antibacterial properties and osteogenic potential of the scaffolds. These approaches offer innovative strategies for designing bone tissue engineering scaffolds capable of regulating immune responses, with the potential to achieve improved clinical outcomes in future therapeutic applications.

## 1 Introduction

In recent years, bone tissue engineering (BTE) has emerged as a significant area of research due to its potential applications in the treatment of bone defects. The primary objective of BTE is to repair or regenerate damaged bone tissue by leveraging the synergistic interactions among biological scaffolds, cells, and bioactive factors (Du et al., 2019). In this methodology, scaffolds function as substitutes for the extracellular matrix (ECM), offering a three-dimensional support structure for osteocytes. They facilitate cell migration, proliferation, and differentiation through specific physical and chemical cues, thereby promoting new bone formation (Zeng et al., 2023; Wei et al., 2024; Xiong et al., 2024). In practical applications, the design of bone tissue engineering scaffolds is progressively advancing towards functionalization. Specifically, the functionality of the scaffold can be enhanced through two primary approaches: first, by altering the chemical composition of the scaffold to achieve targeted functionality; and second, by optimizing the morphology and structure of the scaffold to regulate cellular behavior, thereby promoting cell adhesion and proliferation. Currently, the main materials used for bone scaffolds predominantly include ceramics, polymers, metals, and composite materials. These materials are extensively utilized in bone tissue engineering due to their distinct mechanical properties, biocompatibility, and degradation characteristics (Koons et al., 2020).

As the global population ages rapidly, the proportion of elderly individuals is rising (de Magalhães, 2025). They are more susceptible to falls and fractures due to lower bone density, weaker muscles, and reduced balance (Coughlan and Dockery, 2014; Huang and Huang, 2024). Infectious bone defects pose a challenge in fracture treatment due to their prolonged duration and poor outcomes. Factors like open bone defects, insufficient debridement during graft surgery, and improper aseptic techniques can lead to postoperative infections (Yu et al., 2024). Specifically, the incidence of infection following internal fixation of closed fractures is approximately 1%, whereas for open fractures, it can exceed 15% (Morgenstern et al., 2018). Furthermore, the inherent porous structure of bone implants predisposes them to bacterial colonization and subsequent infection. This is particularly critical in the early stages post-implantation, where pathogenic bacteria can adhere to the surface of the implants prior to the arrival of the body’s immune cells, thereby exerting a head start effect (Chu et al., 2024; Pettygrove et al., 2021). This initial colonization facilitates the formation of a biofilm, which significantly impedes the efficacy of antimicrobial agents in penetrating the barrier. Ultimately, uncontrolled infections may lead to chronic osteomyelitis, characterized by prolonged infection, a high recurrence rate, and a significant disability rate (Spiegel et al., 2010).

Therefore, integrating antimicrobial properties into bone scaffolds holds substantial clinical and scientific importance (Feng et al., 2024; Wang Z. et al., 2025). Currently, a prevalent antibacterial strategy involves utilizing the scaffold as a carrier to deliver antibacterial agents, thereby eradicating bacteria (Zhou et al., 2018; De Silva et al., 2018). However, these biomaterials often prioritize their direct bactericidal effects, potentially at the expense of their role in supporting immune cell defense functions. This oversight may inadvertently compromise the active antibacterial response and timely repair mechanisms of immune cells, potentially resulting in chronic inflammation (Li et al., 2017). Additionally, as foreign implants, scaffolds frequently induce adaptive changes within the host post-implantation *in vivo*. Upon implantation, the scaffold interacts with blood components such as platelets and fibronectin, rapidly initiating an immune response. This immune response subsequently influences tissue repair and the biodegradation process of the scaffold. The interaction of the scaffold with blood components like platelets and fibronectin further prompts the recruitment of immune cells (Scherlinger et al., 2023; Anders and Schaefer, 2014). This process, termed the foreign body reaction, is typically characterized by the recruitment and activation of immune cells, with a particular emphasis on the involvement of neutrophils. Neutrophils serve as a critical “first line of defense” in the initial immune response. Their swift aggregation not only facilitates antibacterial actions but also influences subsequent tissue repair processes. As the main effector cells of an inflammatory response, they can quickly gather at the injured/infected site to remove pathogens and trigger a strong inflammatory response (Kraus and Gruber, 2021). After the inflammatory period, the neutrophils in the injured site gradually subside and initiate the process of tissue repair, and the bone tissue gradually develops in the direction of healing. The implantation of biomaterials to modulate the immune response for tissue repair represents a significant strategy in bone tissue engineering research. Controllable regulation of neutrophil activity via biomaterials is an effective approach to initiating an early immune response, thereby establishing favorable conditions for subsequent osteogenic repair.

This paper provides a comprehensive review of the mechanisms by which neutrophils clear bacteria, including cell phagocytosis, degranulation, the formation of neutrophil extracellular traps (NETs), and the oxidative respiratory burst. Additionally, it explores the role of neutrophils in osteogenic repair. The strategies for regulating neutrophil antibacterial activity and osteogenic repair through scaffolds are introduced, encompassing the loading of metal ions or bioactive factors, the construction of oxygen supply platforms, and the optimization of scaffold morphology and structure. Finally, this review summarizes the related challenges faced by current bone scaffolds in the antibacterial and bone repair process by regulating neutrophils.

## 2 Antibacterial function of neutrophils

**FIGURE 1:**
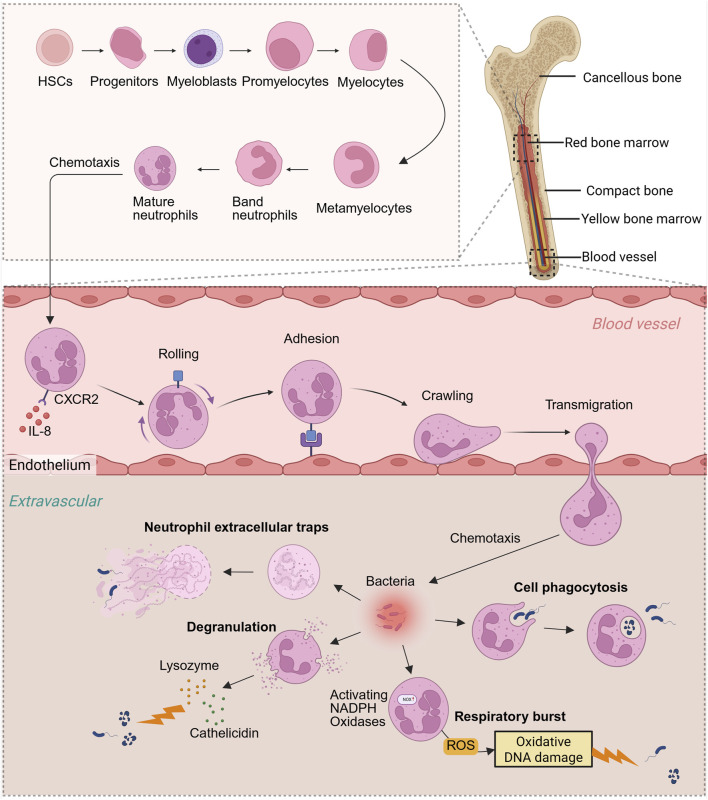
Neutrophil production process and antibacterial function *in vivo*. Neutrophils originate from bone marrow HSCs, progenitors, myeloblasts, promyelocytes, myelocytes, metamyelocytes, band neutrophils, and eventually develop into mature neutrophils in the bone marrow. Neutrophils are chemotaxised by IL-8 in the blood and migrate from the bone marrow to the blood vessels, then “roll,” “adhere” and “crawl,” and finally pass through the blood vessels and enter the outside of the blood vessels. Cytokine chemotaxis subjected to concentration gradient eventually reaches the site of pathogen infection. Neutrophils mainly rely on oxidative respiratory bursts, cell phagocytosis, degranulation, and NETs to fight bacteria. Created with BioRender.com.

Neutrophils originate from myeloid progenitor cells and undergo maturation in the bone marrow, during which their surface receptors transition from CXCR4 to CXCR2. Subsequently, they are released into the bloodstream in response to IL-8-mediated chemotaxis (Németh et al., 2020). Within the blood vessels, neutrophils must extravasate from the vascular bed and migrate to sites of inflammation. This extravasation process comprises four distinct steps: rolling, adhesion, crawling, and transmigration (Li et al., 2024) (Figure 1). When tissues incur damage due to physical, chemical, or biological factors *in vivo*, the affected or necrotic cells release damage-associated molecular patterns (DAMPs) (Huang et al., 2024). These DAMPs activate innate immune cells, prompting the production and release of various chemokines, including CXCL8, CCL2, and CCL5. These chemokines establish concentration gradients around the damaged tissue. Neutrophils, through chemokine receptors on their surfaces such as CXCR1 and CXCR2, detect these gradients and migrate towards the site of damage. Subsequently, neutrophils identify damaged cells or invading microorganisms within tissues and initiate the secretion of signaling molecules, including LTB4 and CXCL2. These signals interact with G protein-coupled receptors on neighboring neutrophils, thereby facilitating the recruitment of additional neutrophil populations (Lämmermann et al., 2013).

**FIGURE 2:**
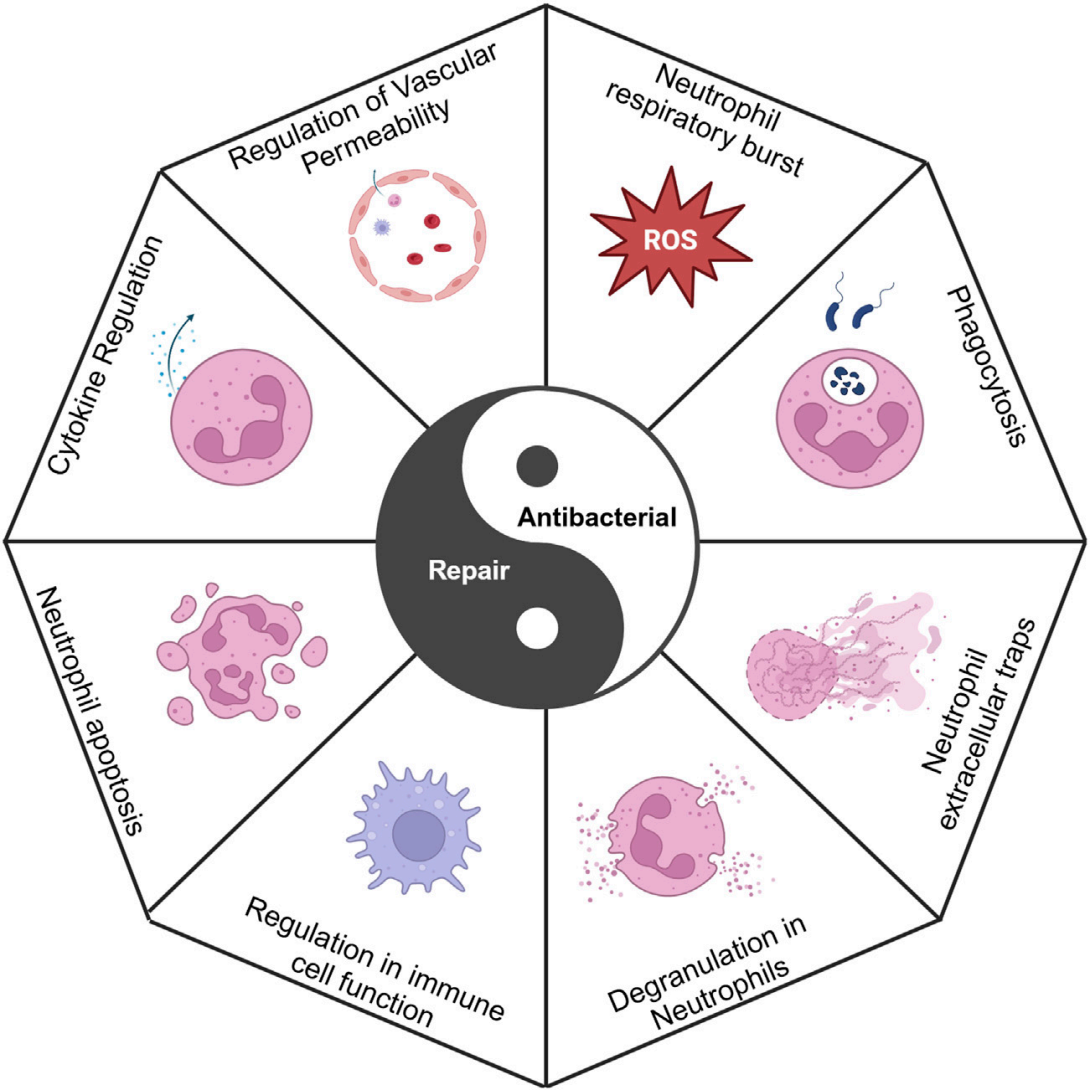
Antibacterial activity and repair maintain a yin-yang balance, in which neutrophils regulate this balance. Created with BioRender.com.

Neutrophils are the first immune cells to infiltrate the site of injury during the initial phase of fracture healing, where they promptly migrate to the affected area to initiate the primary inflammatory response. This inflammatory reaction is crucial for the clearance of damaged tissue, the release of growth factors, and the recruitment of additional immune cell types. The process facilitates the dilation of local blood vessels and enhances blood flow, thereby delivering essential immune cells, nutrients, and oxygen to the site of injury. This initial phase of inflammation is crucial for the removal of damaged tissue, the recruitment of stem cells, and the activation of osteoblasts, all of which are indispensable for fracture repair (Suliman et al., 2022). Nonetheless, excessive or prolonged inflammation can adversely impact the healing process. Excessive inflammation can result in the release of an overabundance of cytokines and enzymes, potentially causing the destruction of surrounding healthy tissue, exacerbating pain and swelling, and disrupting the normal bone healing process. A chronic inflammatory state may lead to delayed or non-union bone healing by persistently activating osteoclasts, thereby increasing bone resorption and weakening newly formed bone tissue. Neutrophil activity plays a crucial role in regulating the balance between inflammation, antibacterial defense, and tissue repair (Figure 2).

### 2.1 Cell phagocytosis

Neutrophils exhibit a phagocytic function, enabling them to directly engulf and eliminate bacterial particles and cellular debris. The neutrophil phagosome, a specialized organelle, is formed through the invagination of the plasma membrane, thereby encapsulating the phagocytosed material. The primary energy source for neutrophils is glycolysis (Toller-Kawahisa et al., 2023), with glucose-driven glycolysis specifically supplying the energy required for neutrophil phagocytosis (Borregaard and Herlin, 1982). Subsequently, intracellular lysosomes fuse with phagosomes to form phagolysosomes. Within these phagolysosomes, microorganisms are eradicated through a combination of oxidative and non-oxidative mechanisms. In the oxidative mechanism, neutrophils undergo a respiratory burst, producing substantial quantities of hydrogen peroxide (H_2_O_2_) (Iyer et al., 1961). Myeloperoxidase (MPO) then utilizes H_2_O_2_ to oxidize chloride ions, generating hypochlorous acid (HOCl), which subsequently damages the bacterial cell membrane and cell wall (Pattison et al., 2012). In the non-oxidative mechanism, neutrophils eliminate microorganisms within the phagolysosome through the release of granule proteins, including MPO, lysozyme, and defensins (Aratani, 2018).

### 2.2 Neutrophil extracellular traps

NETs are structures formed by activated neutrophils that capture and neutralize extracellular pathogens through the release of extracellular trapping components. These components include nuclear materials such as DNA and histones, as well as intracellular granule proteins like neutrophil elastase (NE) and MPO, along with antimicrobial peptides (Wang H. et al., 2024). NETs consist of a DNA backbone intertwined with various proteins, creating a network structure that entraps and restricts the mobility of pathogens. Following entrapment, antibacterial proteins such as MPO and elastase within the NETs can degrade cell walls or cell membranes of the captured pathogens. NETs are categorized into NADPH oxidase (NOX)-dependent and NOX-independent types based on their formation mechanisms. NOX-dependent NETs primarily depend on the activation of NOX to enhance the production of superoxide and reactive oxygen species (ROS). These active substances can induce the degranulation of neutrophil granule proteins, such as MPO and NE, which subsequently cause cytoskeletal rearrangement and nuclear membrane rupture, ultimately resulting in the release of NETs (Feitz et al., 2021). Factors such as lipopolysaccharide (LPS), IL-6, IL-8, TNF-α, various pathogens, and chemicals have been shown to trigger NOX-dependent NETs (Fousert et al., 2020). In contrast, during the formation of NOX-independent NETs, NETs are predominantly released through nuclear membrane blistering and vesicle transport mechanisms (Kaplan and Radic, 2012). Certain bacteria are capable of inducing neutrophils to undergo NOX-dependent NETs. In contrast, *Candida albicans* can stimulate neutrophils to inhibit fungal dissemination through the formation of Dectin-2-mediated, NOX-independent NETs. Additionally, LPS from Gram-negative intracellular bacteria activates a caspase-11-dependent pathway that cleaves the gasdermin D protein, compromising plasma membrane integrity and facilitating the release of NETs (Liang et al., 2021).

### 2.3 Degranulation

Neutrophils encompass a diverse array of granules, which are categorized into primary granules, specific granules, tertiary granules, and secretory vesicles based on their distinct characteristics and constituent substances. Primary granules, in particular, are enriched with the most toxic mediators within the cells, including key components such as MPO, NE, cathepsin G, and defensins. Specific granules contain elements like lactoferrin and lysosomes. Tertiary granules primarily serve as storage sites for metalloproteinases, including gelatinase and leukolysin (Teng et al., 2017). The release of proteases and peptidases into phagolysosomes or through exocytosis to exert antibacterial effects is termed the degranulation process, which is crucial for the non-oxidative sterilization function of neutrophils. NE can directly disrupt the membrane structure of Gram-negative bacteria, such as *Escherichia coli*, leading to bacterial death (Teng et al., 2017). Additionally, lactoferrin not only binds iron, thereby reducing bacterial iron absorption and inhibiting bacterial growth, but also binds to the lipopolysaccharide of the bacterial cell wall, resulting in bacterial oxidation and cleavage (Telang, 2018).

### 2.4 Neutrophil respiratory burst

**FIGURE 3:**
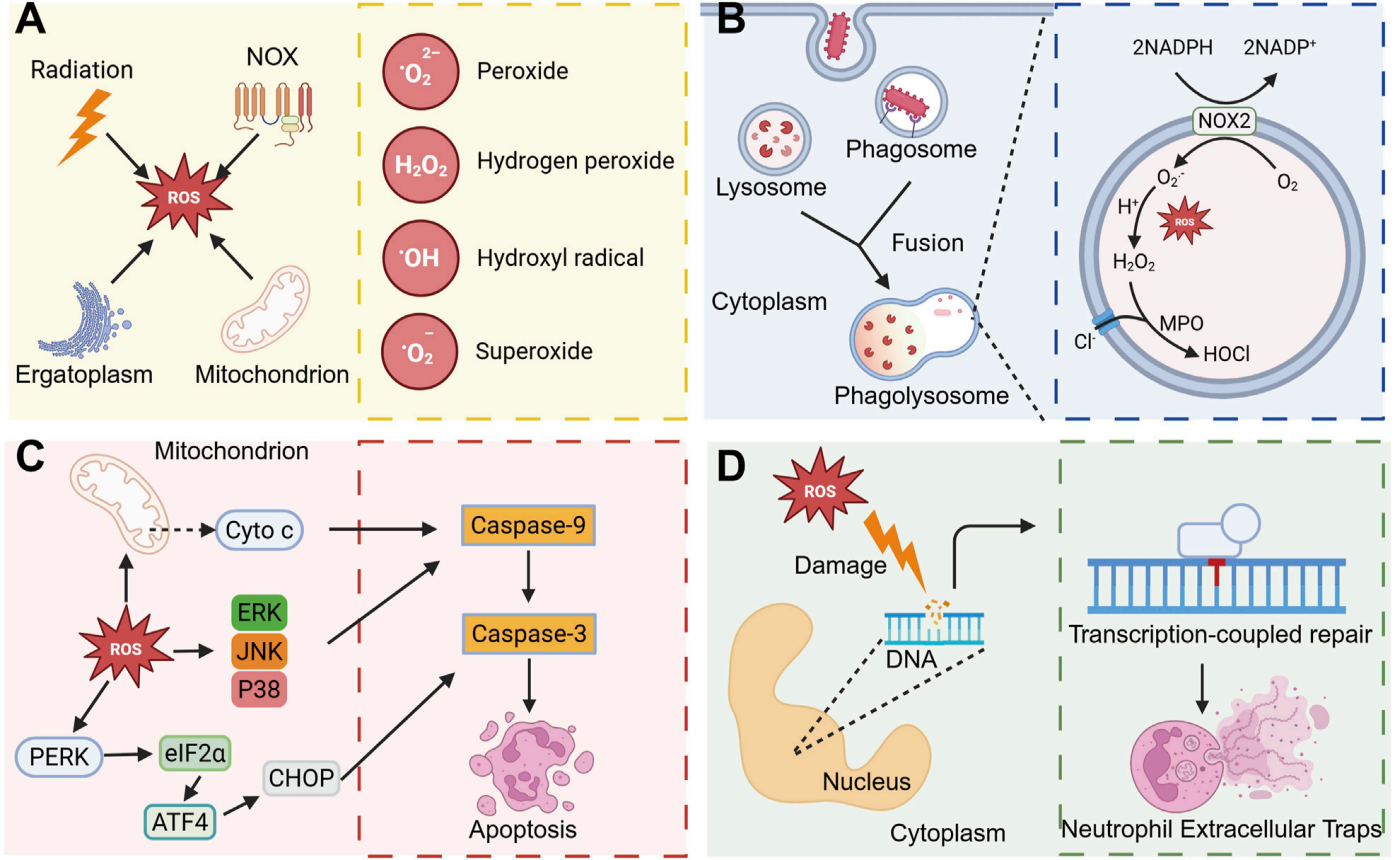
ROS regulates neutrophil activity to regulate inflammatory processes. **(A)** ROS mainly include Peroxide, hydrogen peroxide, Hydroxyl radical, and Superoxide, and these ROS can be produced mainly by ionizing radiation, endoplasmic reticulum stress, NOX, and mitochondria. **(B)** When the pathogen is phagocytosed by neutrophils, phagosomes are formed, and then phagosomes fuse with intracellular lysosomes to form phagolysosomes. In phagolysosomes, ROS produced by NOX2 is converted into H_2_O_2_, and under the action of MPO, it is converted into HOCl with strong antibacterial ability with Cl^−^. **(C)** ROS plays an important role in inducing apoptosis. ROS can activate the MAPK pathway or promote the release of cytochrome c from mitochondria, and then activate Caspase-9 and Caspase-3 and induce apoptosis. ROS is able to activate the PERK-eIF2α-ATF4 signaling pathway and induce apoptosis. **(D)** ROS can cause DNA damage in the nucleus, and can induce the formation of NETs in subsequent DNA repair. Created with BioRender.com.

ROS are predominantly generated as byproducts within the electron transport chain of mitochondria, particularly during the oxidative phosphorylation processes at complex I and complex III (Zhao et al., 2019). In the endoplasmic reticulum, the presence of misfolded proteins or the accumulation of unfolded proteins can disrupt the redox balance and alter Ca^2+^ levels, subsequently inducing ROS production (Ong and Logue, 2023). Ionizing radiation has the capacity to generate ROS, which subsequently target biological macromolecules such as DNA and proteins, leading to cellular damage (Yang et al., 2021) (Figure 3A). Typically, these ROS are intracellularly converted to hydrogen peroxide (H_2_O_2_) by superoxide dismutase and ultimately rendered harmless as H_2_O by catalase (Alfonso-Prieto et al., 2009). However, in neutrophils, the majority of ROS are actively produced through the activation of the nicotinamide adenine dinucleotide phosphate (NADPH) oxidase complex. ROS exhibit significant oxidative potential, capable of inflicting damage on bacterial cell membranes, proteins, and nucleic acids, thereby leading to bacterial death or inactivity (Vatansever et al., 2013).

ROS not only directly eliminates bacteria but also modulates various biological functions of neutrophils, thereby influencing inflammatory processes (Lennicke and Cochemé, 2021). Phagolysosomes, organelles characterized by their oxidative and lytic properties, rely on ROS generated by NADPH oxidase for bacterial eradication (Figure 3B). Furthermore, neutrophil apoptosis plays a crucial role in maintaining homeostasis *in vivo* and facilitates the resolution of inflammation (Karmakar et al., 2021). Excessive production of ROS can result in mitochondrial damage and alterations in mitochondrial membrane potential, subsequently facilitating the release of cytochrome C. This release interacts with apoptotic protease-activating factor 1 to form apoptotic bodies, which then activate cysteine proteases, including caspase-9, thereby initiating the apoptotic pathway (Geering and Simon, 2011). ROS have been demonstrated to activate c-Jun N-terminal kinase and p38 mitogen-activated protein kinase (MAPK), further promoting apoptosis (Zhang et al., 2015). Furthermore, the ROS-mediated PERK-eIF2α-ATF4 pathway is crucial in modulating CHOP-DR5 signaling and subsequent cell apoptosis (Liu and Zhang, 2020) (Figure 3C). Additionally, neutrophil oxidative respiratory bursts are implicated in the formation of NETs. The excessive ROS produced during neutrophil activation leads to significant DNA damage, which subsequently triggers NETs through the DNA repair pathway (Azzouz et al., 2021) (Figure 3D).

## 3 Pro-inflammatory effects of neutrophils

**FIGURE 4:**
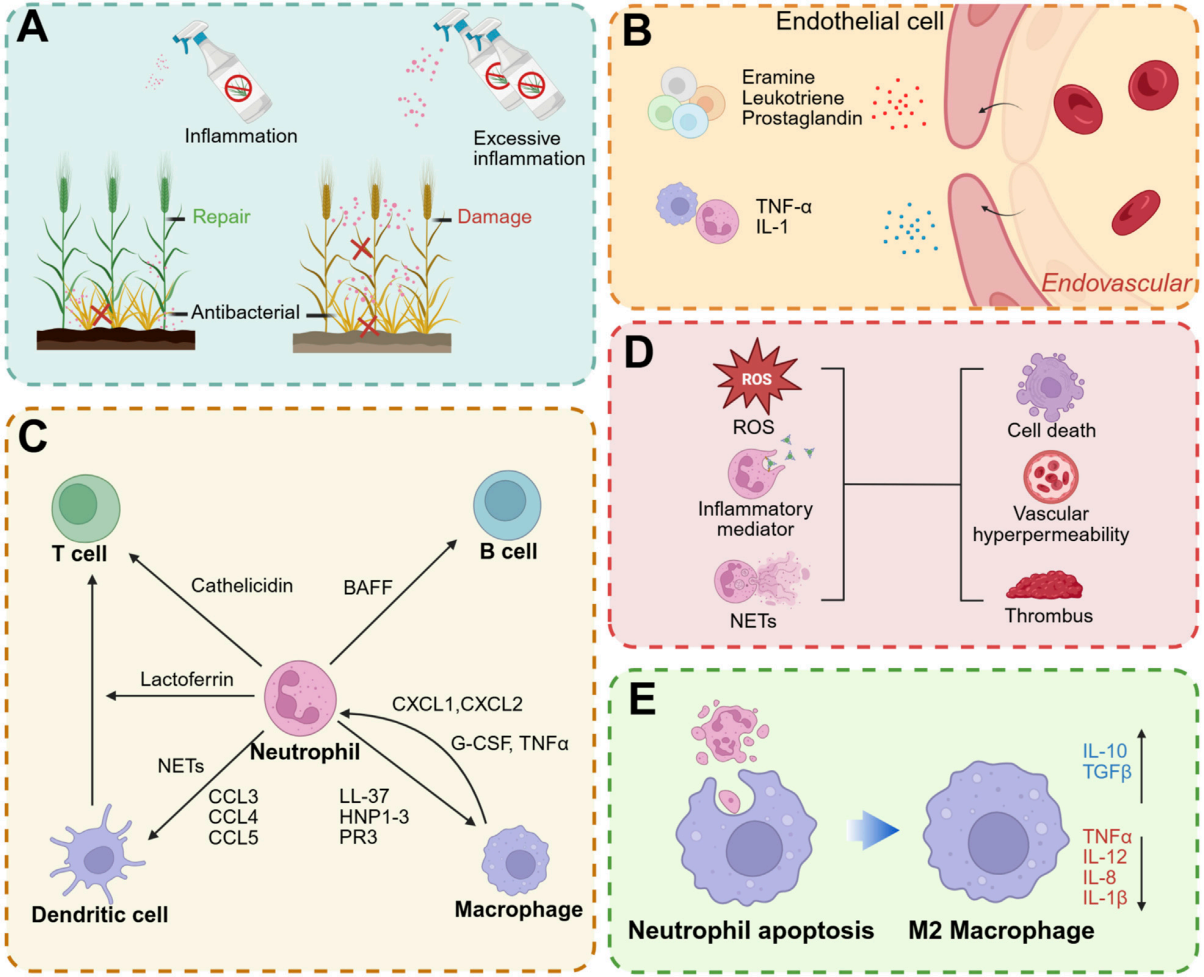
Effect of neutrophils on inflammation. **(A)** An appropriate inflammatory response is beneficial to fight pathogens and promote tissue repair, while an excessive immune response can lead to tissue damage. **(B)** Neutrophils can release inflammatory mediators to promote vascular permeability. **(C)** Interaction between neutrophils and other immune cells. **(D)** Overactivated neutrophils can also cause damage to normal tissues through ROS, inflammatory mediators and NETs. **(E)** After uptake of neutrophil apoptotic bodies, it can promote macrophage polarization to M2 type: anti-inflammatory factors such as IL-10 and TGFβ are upregulated, while pro-inflammatory factors such as TNFα, IL-12, IL-1β and IL-8 are downregulated. Created with BioRender.com.

Neutrophils play a crucial role in mediating inflammatory responses and possess antibacterial capabilities to combat foreign pathogens. However, this inflammatory response can act as a double-edged sword; if not properly regulated, it can result in detrimental effects on host tissues. While an appropriate inflammatory response is beneficial for bacterial resistance and tissue repair, excessive or prolonged inflammation can inflict damage on normal tissues and impede subsequent repair processes (Figure 4A).

### 3.1 Increased vascular permeability and chemotaxis

The initial phase of inflammation is characterized by vasodilation, enhanced blood flow, and increased vascular permeability. The release of histamine and leukotrienes from mast cells induces vasodilation and tissue fluid exudation. Concurrently, neutrophils release inflammatory mediators, including IL-1, IL-6, and TNF-α, which further promote vasodilation (Anzai et al., 2015) and facilitate the extravasation of leukocytes and plasma to the site of injury or infection (Figure 4B). Furthermore, neutrophils generate LTB4 and chemokines, which facilitate the recruitment of additional neutrophils to the site of inflammation (Ng et al., 2011). Upon arrival at the inflammatory site, neutrophils further recruit monocytes by secreting LL-37, cathepsin G, human neutrophil peptides 1-3 (HNP1-3), and protease 3 (PR3), thereby amplifying the inflammatory response (Soehnlein et al., 2008; McDonald et al., 2010).

### 3.2 Affecting immune cells

The immune system comprises a diverse array of immune cells that collaborate to achieve precise mutual regulation through a complex signaling network (Lokwani et al., 2024). As pivotal effector cells within the innate immune system, neutrophils not only directly engage in pathogen clearance but also modulate the function and activity of other immune cells by releasing cytokines, thereby further orchestrating the adaptive immune response (Herrero-Cervera et al., 2022; Scapini and Cassatella, 2014). Neutrophils, lymphocytes, macrophages, dendritic cells (DCs), and other immune cells dynamically interact during the immune response to collectively maintain immune equilibrium and homeostasis (Figure 4C). B cell helper neutrophils localize to the peri-follicular regions of the human spleen under homeostatic conditions, where they express B-cell activating factor. These neutrophils actively contribute to the survival, maturation, and differentiation of B cells, thereby facilitating the development of humoral immunity (Costa et al., 2019; Puga et al., 2011). Neutrophil-derived cathelicidin facilitates the differentiation of T cells towards the Th17 phenotype and enhances their survival (Minns et al., 2021). Soluble mediators secreted by memory T cells, along with direct cell-cell interactions between neutrophils and T cells, collectively promote the acquisition of antigen-presenting capabilities by neutrophils, thereby augmenting their role in adaptive immunity (Lin and Loré, 2017). Furthermore, neutrophils facilitate the recruitment of DCs through the secretion of chemokines such as CCL3, CCL4, CCL5, and CCL20, while the release of NETs stimulates plasmacytoid DCs to secrete inflammatory cytokines (Schuster et al., 2013). The activation of neutrophils to release their granular contents can modulate DC activity and consequently influence T cell function (Hafkamp et al., 2021). Additionally, the secretion of lactoferrin by neutrophils enhances the T cell stimulatory capacity of DCs (de la Rosa et al., 2008). Neutrophils collaborate with macrophages to augment their antibacterial activity (Ladero-Auñon et al., 2021). Macrophages facilitate the recruitment of neutrophils to inflamed sites through the secretion of chemoattractants such as CXCL1 and CXCL2, and they release granulocyte-macrophage colony-stimulating factors (GM-CSF, G-CSF) and TNFα to inhibit apoptosis (Prame Kumar et al., 2018). Simultaneously, neutrophils are capable of releasing LL-37, HNP1-3, and PR3, which serve to attract monocytes and subsequently amplify the inflammatory response. In response to pathogenic challenges, neutrophil-released NETs promote the polarization of macrophages towards the pro-inflammatory M1 phenotype (Tan et al., 2024). Conversely, following neutrophil apoptosis, the resultant apoptotic bodies facilitate the polarization of macrophages towards the anti-inflammatory M2 phenotype (Kraynak et al., 2020; Kraynak et al., 2022).

### 3.3 Damaged tissue

Neutrophils are capable of releasing a diverse array of inflammatory mediators, including proteases, ROS, cytokines, and chemokines. While these mediators play a crucial role in the body’s defense against pathogenic invasion, their excessive release can result in detrimental effects, such as the disruption of tissue architecture and increased vascular permeability (Saffarzadeh et al., 2012). This hyperactivity can further recruit additional immune cells, thereby exacerbating the inflammatory response. Specifically, neutrophil-derived proteases have the potential to degrade the extracellular matrix and compromise cellular structures, culminating in tissue damage (Cartwright et al., 2024). Furthermore, ROS produced by neutrophils can inflict damage on lipids, proteins, and DNA of normal cells, leading to cellular dysfunction and death (Lennicke and Cochemé, 2021; Ma et al., 2024). While NETs are capable of capturing and eliminating pathogens, they also play a role in the pathogenesis of various diseases (Chamardani and Amiritavassoli, 2022; He et al., 2023; Katsoulis et al., 2024; Leffler et al., 2012). Owing to their unique network structure, NETs provide an effective scaffold for thrombosis (Fuchs et al., 2010). The resultant thrombi can exacerbate tissue damage (Le et al., 2022; Thakur et al., 2023; Mereweather et al., 2023) (Figure 4D).

### 3.4 Prolonging the duration of inflammation

The regression of neutrophils from the site of inflammation is primarily mediated through macrophage endocytosis or their return to vascular circulation via reverse migration (de Oliveira et al., 2016; Greenlee-Wacker, 2016). Upon neutrophil apoptosis, low concentrations of nucleotides such as ATP and UTP, which can be released by the cells, act as “Find-me” signals. These nucleotides bind to P2Y2 purinergic receptors on macrophages, facilitating their recognition and subsequent phagocytosis by macrophages (Leist et al., 1997; Elliott et al., 2009). Following apoptosis, the formation of granular shedding or autophagosomes leads to the creation of apoptotic bodies. These apoptotic bodies are primarily composed of extracellular vesicles containing cytoplasm, organelles, and nuclear debris. Subsequently, macrophages phagocytose these apoptotic bodies, a process that promotes macrophage polarization towards the M2 phenotype (Liu et al., 2024). This polarization promotes the production of anti-inflammatory cytokines such as IL-10 and TGFβ, while simultaneously reducing the levels of pro-inflammatory cytokines including TNFα, IL-1β, IL-8 and IL-12 (Fadok et al., 1998; Xu et al., 2025) (Figure 4E). Engineered neutrophil apoptotic bodies mitigate myocardial infarction by facilitating macrophage endocytosis and promoting the resolution of inflammation (Bao et al., 2022). In the absence of rapid clearance, apoptotic neutrophils have the potential to form Gasdermin E pores, activate Peptidylarginine deiminase 4, and induce the release of NETs from apoptotic cells (Zhu et al., 2023).

Neutrophil apoptosis constitutes a fundamental physiological mechanism that facilitates the resolution of inflammation and promotes tissue repair (Karmakar et al., 2021). Nonetheless, the presence of granulocyte-macrophage colony-stimulating factor (GM-CSF) (Yamasawa et al., 2004), Type-1 interferons (Aga et al., 2018), IL-8 (Porter et al., 2017), bacterial components (Miralda et al., 2022), hypoxic conditions (Porter et al., 2017; Dölling et al., 2022), and various other pro-inflammatory mediators can extend neutrophil lifespan, thereby contributing to a sustained inflammatory response. For instance, both *Anaplasma phagocytophilum* and *Chlamydia pneumoniae* have been shown to enhance the phosphorylation of p38 MAPK, activate the PI3K/Akt signaling pathway, and sustain the expression of the anti-apoptotic protein Mcl-1, thereby leading to delayed neutrophil apoptosis (Sarkar et al., 2012; Sarkar et al., 2015). Conversely, hypoxia-inducible factors HIF-1α and HIF-2α can extend the lifespan of neutrophils *in vivo* under hypoxic conditions (Thompso et al., 2014; Walmsley et al., 2005). The inhibition of neutrophil apoptosis consequently promotes the inflammatory response (Wang et al., 2021). The sustained presence and activation of neutrophils in chronic inflammation can perpetuate the inflammatory response, disrupt the equilibrium of tissue repair and regeneration processes, and ultimately result in tissue fibrosis and the onset of other chronic diseases.

## 4 Effect of neutrophils on osteogenesis

**FIGURE 5:**
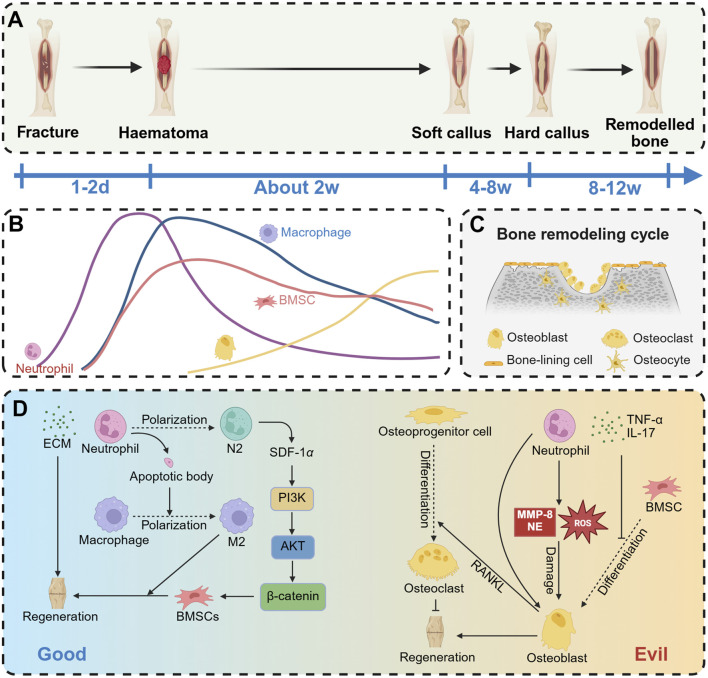
Effect of neutrophils on osteogenesis. **(A)** The process of fracture repair mainly includes hematoma organization phase, callus formation phase and bone remodeling phase. **(B)** Aseptic inflammation is caused by fracture occurrence. Early recruitment of neutrophils to the inflammatory site reaches its peak, followed by a decrease in neutrophil numbers within 1–2 days, and then osteoblasts begin to appear. **(C)** In the late stage of fracture healing, bone remodeling mainly dominates, with osteoblasts and osteoclasts maintaining a delicate balance. **(D)** Neutrophils have two opposing effects on osteogenesis. On the one hand, they can promote ECM secretion to enhance osteogenesis, or facilitate osteogenesis by polarizing into N2 neutrophils or inducing macrophage polarization to M2. On the other hand, neutrophils can promote the differentiation of osteoprogenitor cells into osteoclasts, release inflammatory mediators such as TNFα and IL-17 (which inhibit BMSC differentiation into osteoblasts), or directly damage osteoblasts by releasing MMP-8, NE, or ROS. Created with BioRender.com.

Fracture repair is a multifaceted and systematically regulated dynamic process. Typically, fracture healing is categorized into three distinct stages: the hematoma formation stage, the callus formation stage, and the callus remodeling stage (Figure 5A). Upon the occurrence of a fracture, blood vessels within the bone tissue and bone marrow are disrupted, leading to hemorrhage and the subsequent formation of a hematoma at the fracture site. This hematoma serves as a scaffold for subsequent tissue growth and repair, facilitated by the formation of a fibrin network within the hematoma. The initial phase of local inflammation is characterized by the rapid formation of a hematoma, which serves as a provisional scaffold and actively recruits immune cells, including neutrophils and monocytes, among others (Kolar et al., 2010). The early recruitment of neutrophils is essential for effective fracture repair (Eming et al., 2007; Xing et al., 2010; Gru et al., 1993). Within 12 h following bone injury, neutrophils are recruited to the hematoma site, where they secrete cytokines to attract additional inflammatory cells and initiate the healing cascade (Schmidt-Bleek et al., 2012). Cai et al. (Cai et al., 2021) investigated the effects of IL-8 loaded onto a gelatin sponge, which was subsequently implanted into the thigh muscle pocket of mice. Flow cytometry analysis revealed that neutrophils were the initial cell type recruited to the implantation site, with their numbers peaking on day 1. Recruitment of bone marrow-derived mesenchymal stem cells (BMSCs) commenced on day 2, reaching a maximum between days 4 and 5. Notably, as neutrophil numbers declined, the accelerated recruitment of BMSCs contributed to the enhancement of the bone healing process (Figure 5B).

The bone remodeling cycle comprises three sequential phases: osteoclast-dominated bone resorption, bone reversal mediated by osteoclast-osteoblast interaction, and osteoblast-dominated bone formation and mineralization (Bolamperti et al., 2022) (Figure 5C). Osteoclasts, which originate from myeloid progenitors in the bone marrow, are multinucleated giant cells formed through the fusion of mononuclear macrophages (Takegahara et al., 2024). Under conditions of chronic inflammation, neutrophils have the capacity to induce osteoclast formation (Moonen et al., 2019). NETs facilitate bone erosion in rheumatoid arthritis by augmenting RANKL-induced osteoclastogenesis (Schneider et al., 2024; Guilherme Neto et al., 2023). Neutrophils contribute to the upregulation of RANKL expression by osteoblasts and promote the differentiation of osteoclast progenitor cells into mature osteoclasts (Schneider et al., 2024). This chronic inflammatory stimulus disrupts the homeostasis between osteoblasts and osteoclasts, resulting in bone loss (Kim et al., 2020).

Neutrophils exhibit a dual role in bone repair, encompassing both beneficial and detrimental aspects (Figure 5D). They are crucial in the initial stages of bone healing and regeneration. During the early inflammatory phase, neutrophils secrete pro-inflammatory mediators, pro-angiogenic growth factors, and osteogenic factors, thereby initiating and triggering the bone regeneration cascade (Chung et al., 2006; Shu et al., 2024; Wang L. et al., 2024). Additionally, neutrophils rapidly synthesize fibronectin and extracellular matrix components to promote fracture healing (Bastian et al., 2016). Neutrophils release cytokines, including SDF-1 (Cai et al., 2021; Kitaori et al., 2009), TNF-α (Böcker et al., 2008), and CXCL7 (Almeida et al., 2016), which facilitate the homing of mesenchymal stem cells (MSCs) to fracture healing sites (Wang L. et al., 2024). Additionally, neutrophils upregulate angiogenic markers such as vascular endothelial growth factor (VEGF), CD34, and FGF-2 to promote angiogenesis (Ardi et al., 2009; Herath et al., 2018), a critical process for subsequent bone repair (Zhu et al., 2020; Saberi et al., 2023). Furthermore, neutrophils modulate immune responses to support bone repair processes. Neutrophil apoptotic bodies have been shown to induce macrophage polarization towards the M2 phenotype, thereby facilitating tissue repair (Bao et al., 2022; Martin et al., 2015). Additionally, N2-type neutrophils play a crucial role in directing the recruitment of bone mesenchymal stem cells and initiating bone regeneration (Cai et al., 2021).

Although neutrophils are indispensable defenders during the initial stages of immune response, their prolonged presence can result in persistent inflammation, delayed fracture healing, and additional damage to the organism (Kovach et al., 2015; Ando et al., 2024). Neutrophils exert antibacterial effects by releasing collagenase, elastase, hydrogen peroxide, and hypochlorous acid, which are effective in bacterial eradication. However, these active substances can also inflict damage on tissues and impede the repair process (Bastian et al., 2018). ROS produced by neutrophils have been shown to induce apoptosis in MSCs and osteoblasts (Singh et al., 2012). Additionally, senescent neutrophils secrete significant amounts of granulocalcin, which disrupts the balance between osteogenesis and adipogenesis in BMSCs (Li et al., 2021). Neutrophils also inhibit the synthesis of mineralized extracellular matrix by human bone marrow-derived stromal cells *in vitro* (Bastian et al., 2018). Furthermore, activated neutrophils can both directly and indirectly promote osteoclastogenesis, thereby disrupting bone homeostasis (Ponzetti and Rucci, 2019).

## 5 Implant-driven neutrophil modulation promotes antibacterial activity and bone healing

Although bone scaffolds are biocompatible, their implantation induces an *in vivo* immune response, typically manifesting as an aseptic inflammatory reaction unless subsequently compromised by pathogens (Jhunjhunwala et al., 2015). Neutrophils are pivotal in mediating the aseptic inflammatory response elicited by biomaterials. They act swiftly to establish an acute inflammatory state through mechanisms such as degranulation, chemokine release, and phagocytosis, which are integral to the immediate immune response following biomaterial implantation. Given the pivotal role of neutrophils in aseptic inflammation induced by biomaterials, researchers have initiated investigations into optimizing scaffold design by modulating neutrophil activity. An emerging strategy involves the development of biomaterial scaffolds that can either enhance neutrophil antibacterial functions or regulate immune responses. By employing specific surface modifications, the controlled release of biologically active molecules, or the improvement of morphology and structure, these scaffolds can augment the phagocytic capacity and secretory functions of neutrophils, thereby fostering a more favorable environment for the host to combat infection and promote bone repair at the implantation site (Table 1).

**TABLE 1:** Scaffolds regulate neutrophil immunity to modulate antibacterial and repair strategies.

Strategy	Main components of scaffold	Biological effects produced	References
Oxygen production	TiO_2_, CaO_2_	Providing oxygen to neutrophils promotes the production of ROS and promotes sterilization	Chu et al. (2024)
Carrying oxygen	Oxyhemoglobin	Providing oxygen to neutrophils promotes the production of ROS and promotes sterilization	Zhu et al. (2024)
Equipped with metal ions	Pure Zn	Enhanced the bactericidal ability of peri-implant neutrophils	Peng et al. (2023)
Equipped with metal ions	Mg, PEO-Fe, Zn	Activating the formation of NETs	Li et al. (2022)
Equipped with metal ions	ZnO, Ag	The release of Ag^+^ and Zn^2+^ stimulates immune function to produce a large number of leukocytes and neutrophils	Mao et al. (2017)
Carrying bioactive factors	Mesoporous bioactive glass	TGFβ1 can regulate neutrophil polarization toward the N2 phenotype	Shi et al. (2024)
Drug delivery	Chitosan/polyethylene oxide scaffold	Celecoxib inhibits inflammatory PMNs function	Zheng et al. (2024)
Carrying bioactive factors	Hyaluronic acid, G-CSF	G-CSF enhanced neutrophil recovery after autologous hematopoietic stem cell transplantation	Kerr et al. (2023)
Chemical composition	Stainless steel	Reduced expression of pro-inflammatory cytokines on hydrophilic surfaces of neutrophils	Brodbeck et al. (2003)
Chemical composition	PCL, Laponite	The PCL/LAP nanofibrous membrane promoted anti-inflammatory N2 neutrophil formation, controlled inflammation, and induced M2 macrophage polarization through the immunomodulatory effects of PDLCs	Xu et al. (2023)
Chemical composition	Pure titanium, titanium alloy, stainless steel, polyetheretherketone (PEEK)	Neutrophils produced higher levels of neutrophil elastase, myeloperoxidase, and neutrophil extracellular traps *in vitro* in response to PEEK and stainless steel than neutrophils on Ti or titanium alloy	Avery et al. (2023)
Chemical composition	Glutaraldehyde, Hexamethylenediisocyanate	Glutaraldehyde-crosslinked collagen promotes neutrophil infiltration and activates macrophages for degradation	Ye et al. (2010)
Microchannel	PCL	Layered structure microchannel scaffolds are able to significantly reduce NETs and promote inflammation resolution	Won et al. (2020)
Diameter	Polydioxanone, Collagen type I	Larger fiber diameters reduce NETs	Fetz et al. (2017)
Diameter	Polydioxanone	The smaller fiber diameter promotes the release of NETs	Fetz et al. (2021)
Porosity	*NA*	Enhanced antibacterial ability of neutrophils after passing through smaller pores	Mukhopadhyay et al. (2024)
Stiffness	Polyacrylamide gels	Neutrophil adhesion increases with substrate stiffness	Erpenbeck et al. (2019)
Stiffness	Polydimethylsiloxane (PDMS)	Neutrophils on stiffer PDMS substrates showed more NET formation and greater secretion of pro-inflammatory cytokines and chemokines	Abaricia et al. (2021)
Stiffness	Gelatin methacrylate	Harder matrix promotes neutrophil transition to the N2 phenotype	Jiang et al. (2023)
Surface aperture	Polydioxanone	Larger surface pores decrease the neutrophil extracellular trap immune response	King and Bowlin (2021)
Roughness	Ti	Rough Ti plate surface reduces NETs and decreases the expression of pro-inflammatory factors	Abaricia et al. (2020)
Roughness	Ti	Neutrophils appear round on smooth Ti surface and diffuse-like on rough Ti surface	Campos et al. (2014)

### 5.1 Bioactive factors

**FIGURE 6:**
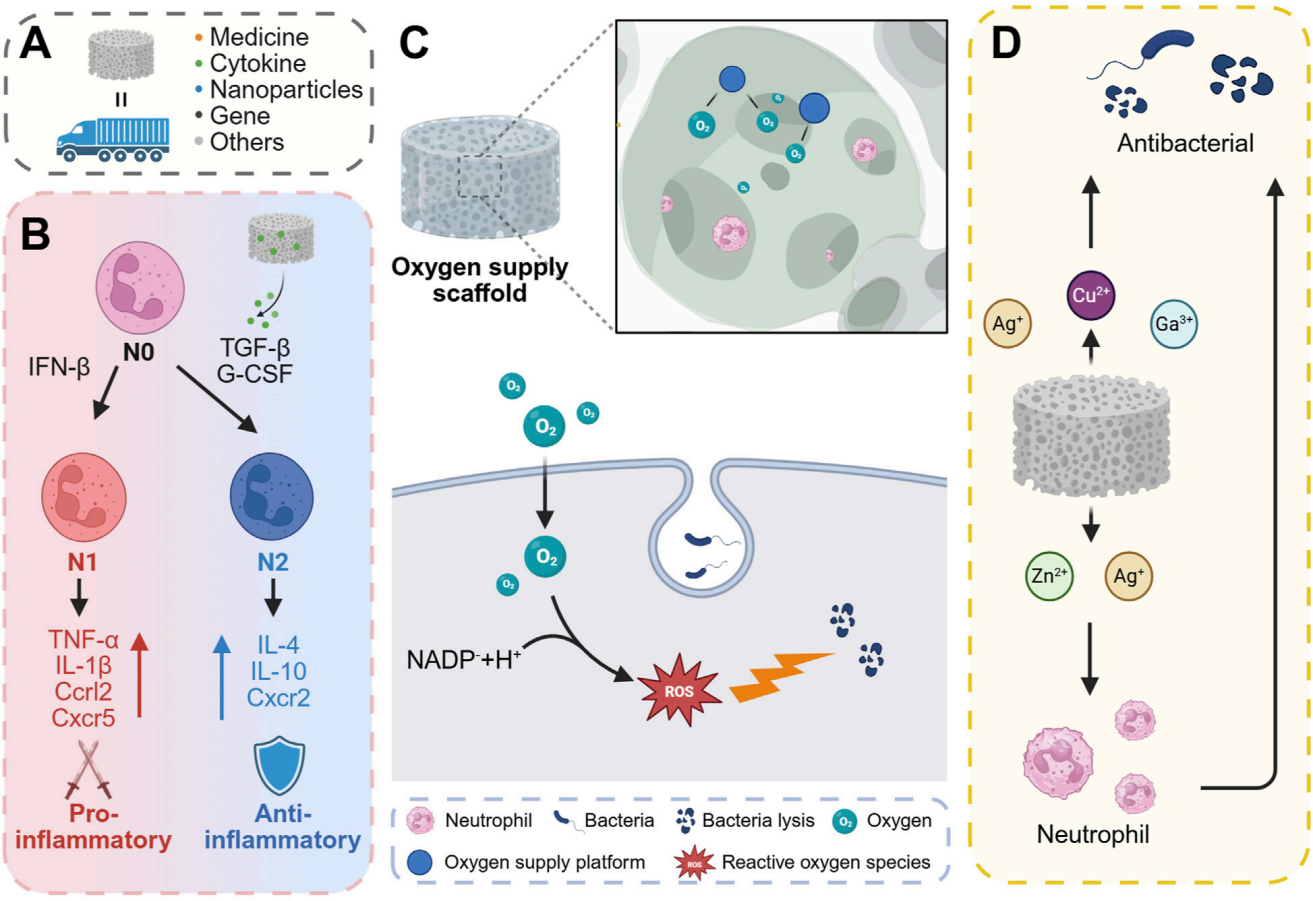
Regulation of neutrophil antibacterial activity or osteogenic function by changing the chemical composition of scaffolds. **(A)** The scaffold can carry drugs, active factors, nanoparticles, genes, and the like as a carrier. **(B)** N0-type neutrophils can be classified as N1 and N2, where N1 is predominantly pro-inflammatory and N2 is predominantly anti-inflammatory. **(C)** The oxygen supply platform in the scaffold can provide oxygen for neutrophil oxidative respiration to promote the antibacterial effect of neutrophils. **(D)** Certain metal ions in the scaffold, such as Ag^+^, Cu^2+^ and Ga^3+^, can directly kill bacteria. At the same time, some metal ions such as Zn^2+^ and Ag^+^ can indirectly kill bacteria by enhancing the bactericidal effect of neutrophils. Created with BioRender.com.

Bone scaffolds serve as carriers capable of incorporating various components, including pharmaceuticals, cytokines, genetic material, and cells, which can be subsequently released at the site of bone defects to fulfill diverse functional roles (Hu et al., 2024) (Figure 6A). The phenotypic switching of neutrophils is regulated by signaling pathways involving TGF-β and IFN-β. Specifically, TGF-β can mediate the conversion of N1 neutrophils to the N2 phenotype (Bu et al., 2022). Exposure of neutrophils to transforming TGF-β or G-CSF results in their polarization towards the N2 phenotype. Conversely, IFN-β has been identified as a pivotal factor in the polarization of neutrophils towards the N1 phenotype in both cancer patients and tumor-bearing mice (Andzinski et al., 2016). N1 neutrophils are capable of upregulating pro-inflammatory mediators such as TNFα, IL-1β, Ccrl2, and Cxcr5. In contrast, N2 neutrophils upregulate anti-inflammatory mediators including IL-4, IL-10, and Cxcr2 (Figure 6B). In a related study, Shi et al. (Shi et al., 2024) developed a genetically engineered composite scaffold by incorporating a hyaluronic acid methacryloyl hydrogel, loaded with a TGF-β1 adenovirus, into a mesoporous bioactive glass scaffold (MBG). Among these, TGF-β1 has been shown to regulate the polarization of neutrophils towards the N2 phenotype and macrophages towards the M2 phenotype through a “relay” mechanism, thereby exerting a reparative effect. Xu et al. (2023) utilized the electrospinning technique to prepare PCL/LAP composites, employing polycaprolactone (PCL) and laponite (LAP) as the constituent materials. The supernatant derived from the co-culture of periodontal ligament cells and a nanofiber membrane has been shown to downregulate the expression of classical pro-inflammatory genes while upregulating the expression of anti-inflammatory and pro-remodeling genes in neutrophils. This suggests a potential role in inducing the polarization of neutrophils towards an anti-inflammatory and pro-remodeling N2 phenotype.

### 5.2 Building oxygen generation and carrying systems

The neutrophil oxidative respiratory burst is crucial for inflammation and antimicrobial activity, consuming large amounts of oxygen to produce superoxide radicals. Activated neutrophils experience increased oxygen consumption during an “oxygen burst” facilitated by NOX2, generating two superoxide radicals per NADPH molecule and two oxygen molecules (Figure 6C). Fractures often damage bone blood vessels, creating a hypoxic microenvironment around the fracture site. Hypoxic conditions can impair ROS synthesis in neutrophils, thereby diminishing their bactericidal capabilities (Beebout et al., 2022). Additionally, hypoxia can inhibit neutrophil apoptosis and extend the inflammatory phase *in vivo* (Rani Talla et al., 2016), which may result in delayed fracture healing (Claes et al., 2012). Consequently, early oxygen supplementation is crucial for both bacterial defense and tissue repair. Enhancing neutrophil antibacterial activity through oxygen delivery has emerged as a strategic approach in the design of effective antibacterial materials. Currently, materials that produce oxygen are utilized to construct oxygen supply platforms. These include the use of solid or liquid peroxides (e.g., CaO_2_, H_2_O_2_) to generate oxygen, or the transportation and release of oxygen via carriers such as fluorinated compounds and erythrocyte membranes. Chu et al. (Chu et al., 2024) introduced an innovative approach by employing *in-situ* deposition of nano-CaO_2_ on the surface of implants to provide localized oxygen support. This method demonstrated a peak oxygen release at 4 h, with sustained release lasting up to 24 h. The CaO_2_/PA-Zn@TiNPs scaffolds were fabricated using a layer-by-layer deposition technique on the surface of TiO_2_ nanopillars, which were pre-coated with a PA-Zn^2+^ coordination complex, followed by the *in-situ* deposition of nano CaO_2_. These scaffolds demonstrated the capability to restore oxygen-dependent neutrophil bactericidal function and support neutrophil ROS production under hypoxic conditions. Additionally, they promoted neutrophil apoptosis, thereby mitigating the subsequent inflammatory response. Zhu et al. (2024) developed a nanomedicine, designated as Hb-Naf@RBCM NPs, by encapsulating red blood cell membrane (RBCM) with nanoparticles formed through the self-assembly of naftifen (Naf) and oxygenated hemoglobin (Hb). The Hb-Naf@RBCM NPs demonstrated the capability to stimulate neutrophils to release superoxide anions and enhance intracellular ROS production, thereby augmenting the neutrophils' response to *Staphylococcus aureus*, multidrug-resistant *S. aureus*, and methicillin-resistant *S. aureus*.

### 5.3 Addition of metal element

Metal ions, including strontium, copper, zinc, silver, magnesium, and iron, are extensively utilized in the design of bone scaffolds due to their ability to regulate the functional balance of osteoblasts and osteoclasts, as well as to promote bone angiogenesis (Luo et al., 2023; Bosch-Rué et al., 2023; O'Neill et al., 2018; Tao et al., 2024; Qian et al., 2021a; Liu et al., 2023; Wang N. et al., 2025). Additionally, these metal ions can influence bacterial metabolic activity and exhibit bactericidal properties (Lemire et al., 2013; Qian et al., 2021b). Specifically, zinc and copper ions have been shown to interfere with bacterial metabolic processes and DNA replication (Wei et al., 2022). Gallium ions (Ga^3+^) interfere with bacterial iron metabolism due to their chemical similarity to iron ions (Fe^3+^), thereby exerting antibacterial and antibiofilm effects (Kaneko et al., 2007). Furthermore, metal nanoparticles, such as those composed of silver and copper, can directly damage bacterial cell walls. These nanoparticles induce the production of ROS, which attack bacterial cell membranes and proteins, ultimately leading to cell death. Additionally, metal ions are crucial components of the body’s immune system (Wang et al., 2020). Metal ions play a pivotal role in regulating various aspects of the immune response and are intricately linked to the pathogenesis and progression of numerous diseases. Specifically, ions such as calcium (Ca), zinc (Zn), manganese (Mn), and magnesium (Mg) are integral to immune signal transduction processes, functioning as second messengers to activate immune cells (Chaigne-Delalande and Lenardo, 2014; Subramanian Vignesh and Deepe, 2016; Krzywoszyńska et al., 2020). Additionally, iron (Fe) and copper (Cu) serve as essential cofactors in the active center of NADPH oxidase, which is critical for the generation of reactive oxygen species (Wu et al., 2020). Iron and zinc chelators have been shown to modulate NETs release (Kuźmicka et al., 2021). Furthermore, an excess of iron, whether due to congenital factors or a high-iron diet, has been found to reduce the release of extracellular traps and reactive oxygen species (Kuźmicka et al., 2022). The release of silver ions (Ag^+^) and zinc ions (Zn^2+^) has been observed to stimulate immune function, leading to an increased production of leukocytes and neutrophils, thereby enhancing antibacterial activity (Mao et al., 2017) (Figure 6D). Pure Zn augmented the bactericidal capacity of peri-implant neutrophils, as evidenced by (Peng et al., 2023). Additionally, Zn plays a pivotal role in mediating the formation of NETs (Hasan et al., 2012). Li et al. (2022) conducted a study where they synthesized Zn-doped FeOOH nanolayers via plasma electrolytic oxidation (PEO) on the surface of a Mg alloy and subsequently coated the alloy with these nanolayers. The incorporation of zinc into the PEO-Fe coating markedly augmented the formation of NETs by diminishing the expression of immune evasion factors and promoting the citrullination of histones and intracellular chromatin depolymerization in neutrophils at the infection site, thereby facilitating NETs formation.

### 5.4 Scaffold structure

**FIGURE 7:**
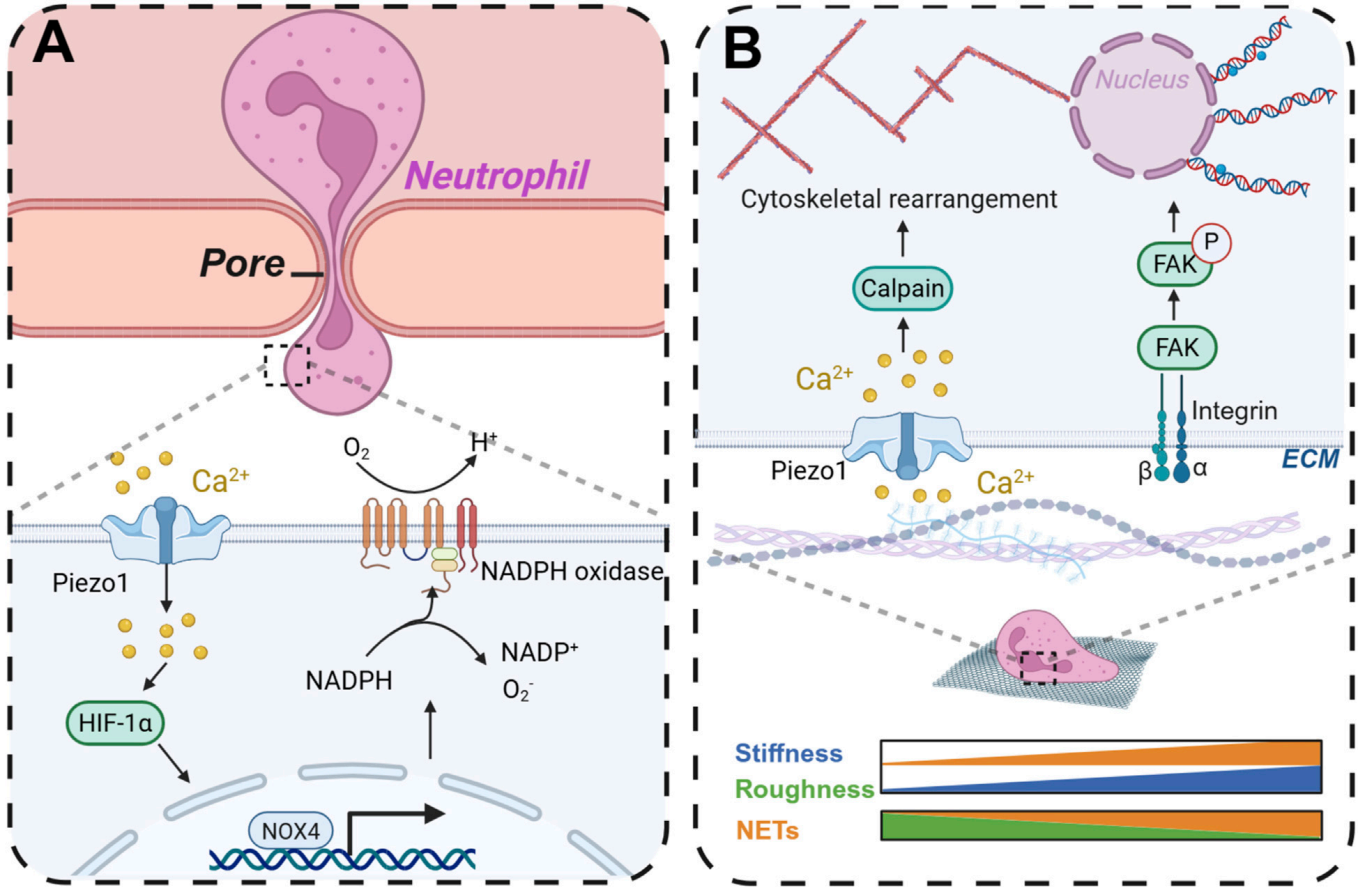
By changing the morphology and structure of scaffolds to regulate neutrophil immunity and thereby modulate antibacterial activity and osteogenic repair. **(A)** When neutrophils cross the pore, they can promote the production of ROS in neutrophils to enhance antibacterial activity. **(B)** The scaffold stiffness and surface morphology affect the production of neutrophil NETs. Created with BioRender.com.

Upon *in vivo* implantation, the scaffold, functioning as an integral component of the extracellular matrix, has the potential to replicate the characteristics of the physiological milieu, thereby influencing cellular behavior (Zhou et al., 2023). The scaffold’s topology, stiffness, and surface topography are critical factors that can modulate neutrophil activity and assume various functional roles. In a study by Won et al. (Won et al., 2020), a hierarchical scaffold incorporating microchannels was fabricated using camphene in a polycaprolactone solution through three-dimensional (3D) printing technology. The internal pore diameter of the scaffold is measured at 12.9 ± 7.69 μm, while the surface pore diameter is 21.1 ± 16.6 μm. Microchannels constitute approximately 28% of the scaffold’s volume. In comparison to 3D printed PCL scaffolds of identical chemical composition but lacking microchannels, the microchannel-incorporated scaffolds demonstrated a significant reduction in NETs, facilitated the resolution of inflammation, and ultimately promoted angiogenesis. Additionally, these scaffolds enhanced stem cell recruitment and chemotaxis, thereby fostering osteogenic differentiation *in vivo*. Allison et al. (Fetz et al., 2017) fabricated electrospun polydioxanone (PDO), type I collagen (COL), and PDO-COL hybrid scaffolds utilizing fibers of small (0.25–0.35 µm) and large (1–2 µm) diameters, observing that larger fiber diameters mitigated the formation of NETs on PDO templates. Furthermore, the pore size of the scaffold can influence the antibacterial efficacy of neutrophils. Mukhopadhyay et al. (2024) demonstrated using a microfluidic device that neutrophils exhibited a significantly enhanced bacterial clearance capability after traversing 5 μm pores compared to 200 μm pores. As neutrophils traverse the pores, their cell membranes experience mechanical stress, which activates the mechanosensor Piezo1. This activation leads to the upregulation of NADPH oxidase 4, thereby augmenting the antibacterial efficacy of polymorphonuclear leukocytes (Figure 7A).

### 5.5 Stiffness

Stiffness denotes the capacity of a material or structure to withstand deformation under the influence of external forces. The stiffness of the ECM is crucial in modulating cellular functions, including viability, communication, migration, and differentiation (Fletcher and Mullins, 2010). Cells can sense ECM stiffness through cell membrane surface receptors and respond accordingly. Additionally, the migration of neutrophils through vascular endothelial cells is influenced by the stiffness of the endothelial cell matrix (Stroka and Aranda-Espinoza, 2011). Neutrophils exhibit reduced migration rates on stiffer substrates but demonstrate enhanced adhesion to rigid surfaces, facilitating their ability to traverse longer distances (Oakes et al., 2009). Abaricia et al. (2021) observed that the formation of NETs was augmented in a stiffness-dependent manner on polydimethylsiloxane (PDMS) substrates with varying physiologically relevant stiffnesses (0.2–32 kPa). Furthermore, the expression of neutrophil chemoattractants and pro-inflammatory factors was elevated on stiffer matrices, a process regulated by integrin/FAK signaling pathways. In a similar vein, Erpenbeck et al. (2019) observed a significant increase in NETs on stiffer polyacrylamide gels following stimulation with LPS, a phenomenon that was attenuated by PI3K inhibition (Figure 7B). Conversely, Jiang et al. (2023) reported that neutrophils exhibited reduced production of ROS and anti-inflammatory cytokines in three-dimensional hydrogel culture systems with higher stiffness. Gelatin methacrylate hydrogels were synthesized with varying degrees of stiffness, revealing that the secretion of pro-inflammatory factors diminished as matrix stiffness increased. Furthermore, the stiffer matrices facilitated the transition of neutrophils to the N2 phenotype, a process regulated by the JAK1/STAT3 signaling pathway.

### 5.6 Surface topography

The interaction between scaffolds and cells is predominantly influenced by the surface topography, which plays a critical role in cell and tissue organization. Surface topography can be engineered on the scaffold through various techniques such as photolithography (Kurland et al., 2014), 3D printing (Liu et al., 2022; Kim et al., 2021), and deposition methods. Upon *in vivo* implantation, the scaffold’s surface topography interfaces with cells, thereby modulating cellular behavior and fate (Rabel et al., 2020; Wang et al., 2012). When cells adhere to various topographical features, they undergo deformation, leading to the activation of mechanoreceptors on the cell membrane. These signals are subsequently transmitted to the nucleus via signaling pathways, ultimately resulting in diverse biological responses (Könnig et al., 2018). Specifically, on smooth titanium (Ti) surfaces, neutrophils adopt a rounded morphology, whereas on rough Ti surfaces, they display a spread-out, flattened morphology (Campos et al., 2014). Modulating the surface topography of the scaffold can influence the cell phenotype, transitioning it from a pro-inflammatory to an anti-inflammatory state. The unique morphological structure of the surface, in response to the shear stress generated by cell membrane contact, can mediate calpain activity and cytoskeletal remodeling through the activation of Piezo1, thereby inducing the production of NETs (Baratchi et al., 2024) (Figure 7B). Neutrophils cultured on rough Ti surfaces exhibited a reduction in the production of pro-inflammatory cytokines and enzymes, as well as a decreased formation of NETs, in comparison to neutrophils on smooth Ti surfaces (Abaricia et al., 2020). This attenuation in NET formation is associated with an accelerated resolution of inflammation and enhanced bone formation on Ti implants (Morandini et al., 2023).

## 6 Conclusion and outlook

In summary, this review primarily focuses on the strategy of neutrophil regulation within bone tissue engineering scaffolds. It aims to modulate non-specific immunity of neutrophils through scaffolds, thereby fostering an immune environment that is favorable for antibacterial activity and tissue repair. In biological organisms, the inflammatory process is typically perceived as a defensive response, whereas the anti-inflammatory process is viewed as a reparative mechanism. Neutrophils are pivotal in regulating both of these processes. Modulating the immune function of neutrophils presents a promising strategy for managing early bacterial infections. Concurrently, neutrophils are also integral to subsequent tissue repair.

Optimizing the physical and chemical properties of scaffolds, regulating the function of neutrophils in antibacterial and osteogenic processes, and achieving a balance between these factors have emerged as significant challenges in scaffold design. While the majority of current tissue engineering research concentrates on either antibacterial efficacy or tissue regeneration, there is a tendency to overlook or inhibit neutrophil activation, or to focus exclusively on eliciting specific macrophage responses. In recent years, the biological function of neutrophils has been progressively elucidated, positioning them as a burgeoning topic in tissue engineering and biomaterials research. Consequently, future investigations into the mechanisms of neutrophils within the biomaterials domain are anticipated to offer novel research avenues for tissue regeneration and immune regulation.

## References

[B1] Abaricia J. O. Shah A. H. Musselman R. M. Olivares-Navarrete R. (2020). Hydrophilic titanium surfaces reduce neutrophil inflammatory response and NETosis. Biomater. Sci. 8 (8), 2289–2299. 10.1039/c9bm01474h 32163073

[B2] Abaricia J. O. Shah A. H. Olivares-Navarrete R. (2021). Substrate stiffness induces neutrophil extracellular trap (NET) formation through focal adhesion kinase activation. Biomaterials 271, 120715. 10.1016/j.biomaterials.2021.120715 33677375 PMC8044006

[B3] Aga E. Mukherjee A. Rane D. More V. Patil T. van Zandbergen G. (2018). Type-1 interferons prolong the lifespan of neutrophils by interfering with members of the apoptotic Cascade. Cytokine 112, 21–26. 10.1016/j.cyto.2018.06.027 30554594

[B4] Alfonso-Prieto M. Biarnés X. Vidossich P. Rovira C. (2009). The molecular mechanism of the catalase reaction. J. Am. Chem. Soc. 131 (33), 11751–11761. 10.1021/ja9018572 19653683

[B5] Almeida C. R. Caires H. R. Vasconcelos D. P. Barbosa M. A. (2016). NAP-2 secreted by human NK cells can stimulate mesenchymal stem/stromal cell recruitment. Stem Cell Rep. 6 (4), 466–473. 10.1016/j.stemcr.2016.02.012 27052313 PMC4834048

[B6] Anders H. J. Schaefer L. (2014). Beyond tissue injury-damage-associated molecular patterns, toll-like receptors, and inflammasomes also drive regeneration and fibrosis. J. Am. Soc. Nephrol. 25 (7), 1387–1400. 10.1681/asn.2014010117 24762401 PMC4073442

[B7] Ando Y. Tsukasaki M. Huynh N.C.-N. Zang S. Yan M. Muro R. (2024). The neutrophil-osteogenic cell axis promotes bone destruction in periodontitis. Int. J. Oral Sci. 16 (1), 18. 10.1038/s41368-023-00275-8 38413562 PMC10899642

[B8] Andzinski L. Kasnitz N. Stahnke S. Wu C.-F. Gereke M. von Köckritz-Blickwede M. (2016). Type I IFNs induce anti-tumor polarization of tumor associated neutrophils in mice and human. Int. J. Cancer 138 (8), 1982–1993. 10.1002/ijc.29945 26619320

[B9] Anzai A. Shimoda M. Endo J. Kohno T. Katsumata Y. Matsuhashi T. (2015). Adventitial CXCL1/G-CSF expression in response to acute aortic dissection triggers local neutrophil recruitment and activation leading to aortic rupture. Circ. Res. 116 (4), 612–623. 10.1161/circresaha.116.304918 25563839

[B10] Aratani Y. (2018). Myeloperoxidase: its role for host defense, inflammation, and neutrophil function. Arch. Biochem. Biophys. 640, 47–52. 10.1016/j.abb.2018.01.004 29336940

[B11] Ardi V. C. Van den Steen P. E. Opdenakker G. Schweighofer B. Deryugina E. I. Quigley J. P. (2009). Neutrophil MMP-9 proenzyme, unencumbered by TIMP-1, undergoes efficient activation *in vivo* and catalytically induces angiogenesis *via* a basic fibroblast growth factor (FGF-2)/FGFR-2 pathway. J. Biol. Chem. 284 (38), 25854–25866. 10.1074/jbc.m109.033472 19608737 PMC2757987

[B12] Avery D. Morandini L. Celt N. Bergey L. Simmons J. Martin R. K. (2023). Immune cell response to orthopedic and craniofacial biomaterials depends on biomaterial composition. Acta Biomater. 161, 285–297. 10.1016/j.actbio.2023.03.007 36905954 PMC10269274

[B13] Azzouz D. Khan M. A. Palaniyar N. (2021). ROS induces NETosis by oxidizing DNA and initiating DNA repair. Cell Death Discov. 7 (1), 113. 10.1038/s41420-021-00491-3 34001856 PMC8128883

[B14] Bao L. Dou G. Tian R. Lv Y. Ding F. Liu S. (2022). Engineered neutrophil apoptotic bodies ameliorate myocardial infarction by promoting macrophage efferocytosis and inflammation resolution. Bioact. Mater 9, 183–197. 10.1016/j.bioactmat.2021.08.008 34820565 PMC8586716

[B15] Baratchi S. Danish H. Chheang C. Zhou Y. Huang A. Lai A. (2024). Piezo1 expression in neutrophils regulates shear-induced NETosis. Nat. Commun. 15 (1), 7023. 10.1038/s41467-024-51211-1 39174529 PMC11341855

[B16] Bastian O. W. Koenderman L. Alblas J. Leenen L. P. H. Blokhuis T. J. (2016). Neutrophils contribute to fracture healing by synthesizing fibronectin+ extracellular matrix rapidly after injury. Clin. Immunol. 164, 78–84. 10.1016/j.clim.2016.02.001 26854617

[B17] Bastian O. W. Croes M. Alblas J. Koenderman L. Leenen L. P. H. Blokhuis T. J. (2018). Neutrophils inhibit synthesis of mineralized extracellular matrix by human bone marrow-derived stromal cells *in vitro* . Front. Immunol. 9, 945. 10.3389/fimmu.2018.00945 29765377 PMC5938347

[B18] Beebout C. J. Robertson G. L. Reinfeld B. I. Blee A. M. Morales G. H. Brannon J. R. (2022). Uropathogenic *Escherichia coli* subverts mitochondrial metabolism to enable intracellular bacterial pathogenesis in urinary tract infection. Nat. Microbiol. 7 (9), 1348–1360. 10.1038/s41564-022-01205-w 35995841 PMC9756876

[B19] Böcker W. Docheva D. Prall W. C. Egea V. Pappou E. Rossmann O. (2008). IKK-2 is required for TNF-alpha-induced invasion and proliferation of human mesenchymal stem cells. J. Mol. Med. Berl. 86 (10), 1183–1192. 10.1007/s00109-008-0378-3 18600306

[B20] Bolamperti S. Villa I. Rubinacci A. (2022). Bone remodeling: an operational process ensuring survival and bone mechanical competence. Bone Res. 10 (1), 48. 10.1038/s41413-022-00219-8 35851054 PMC9293977

[B21] Borregaard N. Herlin T. (1982). Energy metabolism of human neutrophils during phagocytosis. J. Clin. Invest 70 (3), 550–557. 10.1172/jci110647 7107894 PMC370256

[B22] Bosch-Rué È. Díez-Tercero L. Buitrago J. O. Castro E. Pérez R. A. (2023). Angiogenic and immunomodulation role of ions for initial stages of bone tissue regeneration. Acta Biomater. 166, 14–41. 10.1016/j.actbio.2023.06.001 37302735

[B23] Brodbeck W. G. Voskerician G. Ziats N. P. Nakayama Y. Matsuda T. Anderson J. M. (2003). *In vivo* leukocyte cytokine mRNA responses to biomaterials are dependent on surface chemistry. J. Biomed. Mater Res. A 64 (2), 320–329. 10.1002/jbm.a.10425 12522819

[B24] Bu M. T. Chandrasekhar P. Ding L. Hugo W. (2022). The roles of TGF-β and VEGF pathways in the suppression of antitumor immunity in melanoma and other solid tumors. Pharmacol. Ther. 240, 108211. 10.1016/j.pharmthera.2022.108211 35577211 PMC10956517

[B25] Cai B. Lin D. Li Y. Wang L. Xie J. Dai T. (2021). N2-Polarized neutrophils guide bone mesenchymal stem cell recruitment and initiate bone regeneration: a missing piece of the bone regeneration puzzle. Adv. Sci. (Weinh) 8 (19), e2100584. 10.1002/advs.202100584 34382372 PMC8498914

[B26] Campos V. Melo R. C. N. Silva L. P. Aquino E. N. Castro M. S. Fontes W. (2014). Characterization of neutrophil adhesion to different titanium surfaces. Bull. Mater. Sci. 37 (1), 157–166. 10.1007/s12034-014-0611-3

[B27] Cartwright I. M. Zhou L. Koch S. D. Welch N. Zakharov D. Callahan R. (2024). Chlorination of epithelial tight junction proteins by neutrophil myeloperoxidase promotes barrier dysfunction and mucosal inflammation. JCI Insight 9 (14), e178525. 10.1172/jci.insight.178525 39133648 PMC11383587

[B28] Chaigne-Delalande B. Lenardo M. J. (2014). Divalent cation signaling in immune cells. Trends Immunol. 35 (7), 332–344. 10.1016/j.it.2014.05.001 24932518 PMC4790094

[B29] Chamardani T. M. Amiritavassoli S. (2022). Inhibition of NETosis for treatment purposes: friend or foe? Mol. Cell Biochem. 477 (3), 673–688. 10.1007/s11010-021-04315-x 34993747 PMC8736330

[B30] Chu G. Guan M. Jin J. Luo Y. Luo Z. Shi T. (2024). Mechanochemically reprogrammed interface orchestrates neutrophil bactericidal activity and apoptosis for preventing implant-associated infection. Adv. Mater 36 (16), e2311855. 10.1002/adma.202311855 38164817

[B31] Chung R. Cool J. C. Scherer M. A. Foster B. K. Xian C. J. (2006). Roles of neutrophil-mediated inflammatory response in the bony repair of injured growth plate cartilage in young rats. J. Leukoc. Biol. 80 (6), 1272–1280. 10.1189/jlb.0606365 16959896

[B32] Claes L. Recknagel S. Ignatius A. (2012). Fracture healing under healthy and inflammatory conditions. Nat. Rev. Rheumatol. 8 (3), 133–143. 10.1038/nrrheum.2012.1 22293759

[B33] Costa S. Bevilacqua D. Cassatella M. A. Scapini P. (2019). Recent advances on the crosstalk between neutrophils and B or T lymphocytes. Immunology 156 (1), 23–32. 10.1111/imm.13005 30259972 PMC6283649

[B34] Coughlan T. Dockery F. (2014). Osteoporosis and fracture risk in older people. Clin. Med. (Lond) 14 (2), 187–191. 10.7861/clinmedicine.14-2-187 24715132 PMC4953292

[B35] de la Rosa G. Yang D. Tewary P. Varadhachary A. Oppenheim J. J. (2008). Lactoferrin acts as an alarmin to promote the recruitment and activation of APCs and antigen-specific immune responses. J. Immunol. 180 (10), 6868–6876. 10.4049/jimmunol.180.10.6868 18453607 PMC2408856

[B36] de Magalhães J. P. (2025). An overview of contemporary theories of ageing. Nat. Cell Biol. 27 (7), 1074–1082. 10.1038/s41556-025-01698-7 40595368

[B37] de Oliveira S. Rosowski E. E. Huttenlocher A. (2016). Neutrophil migration in infection and wound repair: going forward in reverse. Nat. Rev. Immunol. 16 (6), 378–391. 10.1038/nri.2016.49 27231052 PMC5367630

[B38] De Silva R. T. Dissanayake R. K. Mantilaka M. M. M. G. P. G. Wijesinghe W. P. S. L. Kaleel S. S. Premachandra T. N. (2018). Drug-loaded halloysite nanotube-reinforced electrospun alginate-based nanofibrous scaffolds with sustained antimicrobial protection. ACS Appl. Mater Interfaces 10 (40), 33913–33922. 10.1021/acsami.8b11013 30220194

[B39] Dölling M. Eckstein M. Singh J. Schauer C. Schoen J. Shan X. (2022). Hypoxia promotes neutrophil survival after acute myocardial infarction. Front. Immunol. 13, 726153. 10.3389/fimmu.2022.726153 35222361 PMC8873092

[B40] Du Y. Guo J. L. Wang J. Mikos A. G. Zhang S. (2019). Hierarchically designed bone scaffolds: from internal cues to external stimuli. Biomaterials 218, 119334. 10.1016/j.biomaterials.2019.119334 31306826 PMC6663598

[B41] Elliott M. R. Chekeni F. B. Trampont P. C. Lazarowski E. R. Kadl A. Walk S. F. (2009). Nucleotides released by apoptotic cells act as a find-me signal to promote phagocytic clearance. Nature 461 (7261), 282–286. 10.1038/nature08296 19741708 PMC2851546

[B42] Eming S. A. Krieg T. Davidson J. M. (2007). Inflammation in wound repair: molecular and cellular mechanisms. J. Invest Dermatol 127 (3), 514–525. 10.1038/sj.jid.5700701 17299434

[B43] Erpenbeck L. Gruhn A. L. Kudryasheva G. Günay G. Meyer D. Busse J. (2019). Effect of adhesion and substrate elasticity on neutrophil extracellular trap formation. Front. Immunol. 10, 2320. 10.3389/fimmu.2019.02320 31632402 PMC6781793

[B44] Fadok V. A. Bratton D. L. Konowal A. Freed P. W. Westcott J. Y. Henson P. M. (1998). Macrophages that have ingested apoptotic cells *in vitro* inhibit proinflammatory cytokine production through autocrine/paracrine mechanisms involving TGF-beta, PGE2, and PAF. J. Clin. Invest 101 (4), 890–898. 10.1172/jci1112 9466984 PMC508637

[B45] Feitz W. J. C. Suntharalingham S. Khan M. Ortiz-Sandoval C. G. Palaniyar N. van den Heuvel L. P. (2021). Shiga toxin 2a induces NETosis *via* NOX-dependent pathway. Biomedicines 9 (12), 1807. 10.3390/biomedicines9121807 34944623 PMC8698832

[B46] Feng P. He R. Gu Y. Yang F. Pan H. Shuai C. (2024). Construction of antibacterial bone implants and their application in bone regeneration. Mater. Horizons 11 (3), 590–625. 10.1039/d3mh01298k 38018410

[B47] Fetz A. E. Neeli I. Rodriguez I. A. Radic M. Z. Bowlin G. L. (2017). Electrospun template architecture and composition regulate neutrophil NETosis *in vitro* and *in vivo* . Tissue Eng. Part A 23 (19-20), 1054–1063. 10.1089/ten.tea.2016.0452 28068879

[B48] Fetz A. E. Radic M. Z. Bowlin G. L. (2021). Human neutrophil FcγRIIIb regulates neutrophil extracellular trap release in response to electrospun polydioxanone biomaterials. Acta Biomater. 130, 281–290. 10.1016/j.actbio.2021.06.007 34116225 PMC8316391

[B49] Fletcher D. A. Mullins R. D. (2010). Cell mechanics and the cytoskeleton. Nature 463 (7280), 485–492. 10.1038/nature08908 20110992 PMC2851742

[B50] Fousert E. Toes R. Desai J. (2020). Neutrophil extracellular traps (NETs) take the central stage in driving autoimmune responses. Cells 9 (4), 915. 10.3390/cells9040915 32276504 PMC7226846

[B51] Fuchs T. A. Brill A. Duerschmied D. Schatzberg D. Monestier M. Myers D. D. (2010). Extracellular DNA traps promote thrombosis. Proc. Natl. Acad. Sci. U. S. A. 107 (36), 15880–15885. 10.1073/pnas.1005743107 20798043 PMC2936604

[B52] Geering B. Simon H. U. (2011). Peculiarities of cell death mechanisms in neutrophils. Cell Death Differ. 18 (9), 1457–1469. 10.1038/cdd.2011.75 21637292 PMC3178425

[B53] Greenlee-Wacker M. C. (2016). Clearance of apoptotic neutrophils and resolution of inflammation. Immunol. Rev. 273 (1), 357–370. 10.1111/imr.12453 27558346 PMC5000862

[B54] Grundnes O. Reikerås O. (1993). The importance of the hematoma for fracture healing in rats. Acta Orthop. Scand. 64 (3), 340–342. 10.3109/17453679308993640 8322595

[B55] Guilherme Neto J. L. Rodrigues Venturini L. G. Schneider A. H. Taira T. M. Duffles Rodrigues L. F. Veras F. P. (2023). Neutrophil extracellular traps aggravate apical periodontitis by stimulating osteoclast formation. J. Endod. 49 (11), 1514–1521. 10.1016/j.joen.2023.07.027 37619708

[B56] Hafkamp F. M. J. Groot Kormelink T. de Jong E. C. (2021). Targeting DCs for tolerance induction: don't lose sight of the neutrophils. Front. Immunol. 12, 732992. 10.3389/fimmu.2021.732992 34675923 PMC8523850

[B57] Hasan R. Rink L. Haase H. (2012). Zinc signals in neutrophil granulocytes are required for the formation of neutrophil extracellular traps. Innate Immun. 19 (3), 253–264. 10.1177/1753425912458815 23008348

[B58] He L. Liu R. Yue H. Zhang X. Pan X. Sun Y. (2023). Interaction between neutrophil extracellular traps and cardiomyocytes contributes to atrial fibrillation progression. Signal Transduct. Target Ther. 8 (1), 279. 10.1038/s41392-023-01497-2 37491321 PMC10368710

[B59] Herath T. D. K. Larbi A. Teoh S. H. Kirkpatrick C. J. Goh B. T. (2018). Neutrophil-mediated enhancement of angiogenesis and osteogenesis in a novel triple cell co-culture model with endothelial cells and osteoblasts. J. Tissue Eng. Regen. Med. 12 (2), e1221–e1236. 10.1002/term.2521 28715156

[B60] Herrero-Cervera A. Soehnlein O. Kenne E. (2022). Neutrophils in chronic inflammatory diseases. Cell Mol. Immunol. 19 (2), 177–191. 10.1038/s41423-021-00832-3 35039631 PMC8803838

[B61] Hu Y. Tang L. Wang Z. Yan H. Yi X. Wang H. (2024). Inducing *in situ* M2 macrophage polarization to promote the repair of bone defects *via* scaffold-mediated sustained delivery of luteolin. J. Control Release 365, 889–904. 10.1016/j.jconrel.2023.11.015 37952829

[B62] Huang A. A. Huang S. Y. (2024). The impact of aging on outcomes in acute respiratory distress syndrome: a multicenter cohort study. Aging Adv. 1 (2), 61–68. 10.4103/agingadv.agingadv-d-24-00024

[B63] Huang Y. Jiang W. Zhou R. (2024). DAMP sensing and Sterile inflammation: intracellular, intercellular and inter-organ pathways. Nat. Rev. Immunol. 24, 703–719. 10.1038/s41577-024-01027-3 38684933

[B64] Iyer G. Y. N. Islam M. F. Quastel J. H. (1961). Biochemical aspects of phagocytosis. Nature 192 (4802), 535–541. 10.1038/192535a0

[B65] Jhunjhunwala S. Aresta-DaSilva S. Tang K. Alvarez D. Webber M. J. Tang B. C. (2015). Neutrophil responses to sterile implant materials. PLoS One 10 (9), e0137550. 10.1371/journal.pone.0137550 26355958 PMC4565661

[B66] Jiang T. Tang X.-Y. Mao Y. Zhou Y.-Q. Wang J.-J. Li R.-M. (2023). Matrix mechanics regulate the polarization state of bone marrow-derived neutrophils through the JAK1/STAT3 signaling pathway. Acta Biomater. 168, 159–173. 10.1016/j.actbio.2023.07.012 37467837

[B67] Kaneko Y. Thoendel M. Olakanmi O. Britigan B. E. Singh P. K. (2007). The transition metal gallium disrupts *Pseudomonas aeruginosa* iron metabolism and has antimicrobial and antibiofilm activity. J. Clin. Invest 117 (4), 877–888. 10.1172/jci30783 17364024 PMC1810576

[B68] Kaplan M. J. Radic M. (2012). Neutrophil extracellular traps: double-edged swords of innate immunity. J. Immunol. 189 (6), 2689–2695. 10.4049/jimmunol.1201719 22956760 PMC3439169

[B69] Karmakar U. Chu J. Y. Sundaram K. Astier A. L. Garside H. Hansen C. G. (2021). Immune complex-induced apoptosis and concurrent immune complex clearance are anti-inflammatory neutrophil functions. Cell Death Dis. 12 (4), 296. 10.1038/s41419-021-03528-8 33741905 PMC7979711

[B70] Katsoulis O. Toussaint M. Jackson M. M. Mallia P. Footitt J. Mincham K. T. (2024). Neutrophil extracellular traps promote immunopathogenesis of virus-induced COPD exacerbations. Nat. Commun. 15 (1), 5766. 10.1038/s41467-024-50197-0 38982052 PMC11233599

[B71] Kerr M. D. McBride D. A. Johnson W. T. Chumber A. K. Najibi A. J. Seo B. R. (2023). Immune-responsive biodegradable scaffolds for enhancing neutrophil regeneration. Bioeng. Transl. Med. 8 (1), e10309. 10.1002/btm2.10309 36684088 PMC9842036

[B72] Kim J.-M. Lin C. Stavre Z. Greenblatt M. B. Shim J.-H. (2020). Osteoblast-osteoclast communication and bone homeostasis. Cells 9 (9), 2073. 10.3390/cells9092073 32927921 PMC7564526

[B73] Kim H.-S. Lee J.-H. Mandakhbayar N. Jin G.-Z. Kim S.-J. Yoon J.-Y. (2021). Therapeutic tissue regenerative nanohybrids self-assembled from bioactive inorganic core/chitosan shell nanounits. Biomaterials 274, 120857. 10.1016/j.biomaterials.2021.120857 33965799

[B74] King W. E. Bowlin G. L. (2021). Mechanical characterization and neutrophil NETs response of a novel hybrid geometry polydioxanone near-field electrospun scaffold. Biomed. Mater 16 (6), 065002. 10.1088/1748-605x/ac1e43 34404034

[B75] Kitaori T. Ito H. Schwarz E. M. Tsutsumi R. Yoshitomi H. Oishi S. (2009). Stromal cell-derived factor 1/CXCR4 signaling is critical for the recruitment of mesenchymal stem cells to the fracture site during skeletal repair in a mouse model. Arthritis Rheum. 60 (3), 813–823. 10.1002/art.24330 19248097

[B76] Kolar P. Schmidt-Bleek K. Schell H. Gaber T. Toben D. Schmidmaier G. (2010). The early fracture hematoma and its potential role in fracture healing. Tissue Eng. Part B Rev. 16 (4), 427–434. 10.1089/ten.teb.2009.0687 20196645

[B77] Könnig D. Herrera A. Duda G. N. Petersen A. (2018). Mechanosensation across borders: fibroblasts inside a macroporous scaffold sense and respond to the mechanical environment beyond the scaffold walls. J. Tissue Eng. Regen. Med. 12 (1), 265–275. 10.1002/term.2410 28084698

[B78] Koons G. L. Diba M. Mikos A. G. (2020). Materials design for bone-tissue engineering. Nat. Rev. Mater. 5 (8), 584–603. 10.1038/s41578-020-0204-2

[B79] Kovach T. K. Dighe A. S. Lobo P. I. Cui Q. (2015). Interactions between MSCs and immune cells: implications for bone healing. J. Immunol. Res. 2015, 1–17. 10.1155/2015/752510 26000315 PMC4427002

[B80] Kraus R. F. Gruber M. A. (2021). Neutrophils-from bone marrow to first-line defense of the innate immune system. Front. Immunol. 12, 767175. 10.3389/fimmu.2021.767175 35003081 PMC8732951

[B81] Kraynak C. A. Yan D. J. Suggs L. J. (2020). Modulating inflammatory macrophages with an apoptotic body-inspired nanoparticle. Acta Biomater. 108, 250–260. 10.1016/j.actbio.2020.03.041 32251779

[B82] Kraynak C. A. Huang W. Bender E. C. Wang J.-L. Hanafy M. S. Cui Z. (2022). Apoptotic body-inspired nanoparticles target macrophages at sites of inflammation to support an anti-inflammatory phenotype shift. Int. J. Pharm. 618, 121634. 10.1016/j.ijpharm.2022.121634 35247497 PMC9007911

[B83] Krzywoszyńska K. Witkowska D. Swiatek-Kozlowska J. Szebesczyk A. Kozłowski H. (2020). General aspects of metal ions as signaling agents in health and disease. Biomolecules 10 (10), 1417. 10.3390/biom10101417 33036384 PMC7600656

[B84] Kurland N. E. Dey T. Wang C. Kundu S. C. Yadavalli V. K. (2014). Silk protein lithography as a route to fabricate sericin microarchitectures. Adv. Mater 26 (26), 4431–4437. 10.1002/adma.201400777 24737390

[B85] Kuźmicka W. Moskalik A. Manda-Handzlik A. Demkow U. Wachowska M. Ciepiela O. (2021). Influence of iron- and zinc-chelating agents on neutrophil extracellular trap formation. Cent. Eur. J. Immunol. 46 (2), 135–139. 10.5114/ceji.2021.106985 34764782 PMC8568028

[B86] Kuźmicka W. Manda-Handzlik A. Mroczek A. Cieloch A. Moskalik A. Demkow U. (2022). Iron excess affects release of neutrophil extracellular traps and reactive oxygen species but does not influence other functions of neutrophils. Immunol. Cell Biol. 100 (2), 87–100. 10.1111/imcb.12509 34714958

[B87] Ladero-Auñon I. Molina E. Holder A. Kolakowski J. Harris H. Urkitza A. (2021). Bovine neutrophils release extracellular traps and cooperate with macrophages in *Mycobacterium avium* subsp. paratuberculosis clearance *in vitro* . Front. Immunol. 12, 645304. 10.3389/fimmu.2021.645304 33815401 PMC8010319

[B88] Lämmermann T. Afonso P. V. Angermann B. R. Wang J. M. Kastenmüller W. Parent C. A. (2013). Neutrophil swarms require LTB4 and integrins at sites of cell death *in vivo* . Nature 498 (7454), 371–375. 10.1038/nature12175 23708969 PMC3879961

[B89] Leung H. H. L. Perdomo J. Ahmadi Z. Zheng S. S. Rashid F. N. Enjeti A. (2022). NETosis and thrombosis in vaccine-induced immune thrombotic thrombocytopenia. Nat. Commun. 13 (1), 5206. 10.1038/s41467-022-32946-1 36064843 PMC9441824

[B90] Leffler J. Martin M. Gullstrand B. Tydén H. Lood C. Truedsson L. (2012). Neutrophil extracellular traps that are not degraded in systemic lupus erythematosus activate complement exacerbating the disease. J. Immunol. 188 (7), 3522–3531. 10.4049/jimmunol.1102404 22345666

[B91] Leist M. Single B. Castoldi A. F. Kühnle S. Nicotera P. (1997). Intracellular adenosine triphosphate (ATP) concentration: a switch in the decision between apoptosis and necrosis. J. Exp. Med. 185 (8), 1481–1486. 10.1084/jem.185.8.1481 9126928 PMC2196283

[B92] Lemire J. A. Harrison J. J. Turner R. J. (2013). Antimicrobial activity of metals: mechanisms, molecular targets and applications. Nat. Rev. Microbiol. 11 (6), 371–384. 10.1038/nrmicro3028 23669886

[B93] Lennicke C. Cochemé H. M. (2021). Redox metabolism: ROS as specific molecular regulators of cell signaling and function. Mol. Cell 81 (18), 3691–3707. 10.1016/j.molcel.2021.08.018 34547234

[B94] Li J. Tan L. Liu X. Cui Z. Yang X. Yeung K. W. K. (2017). Balancing bacteria-osteoblast competition through selective physical puncture and biofunctionalization of ZnO/Polydopamine/arginine-glycine-aspartic acid-cysteine nanorods. ACS Nano 11 (11), 11250–11263. 10.1021/acsnano.7b05620 29049874

[B95] Li C.-J. Xiao Y. Sun Y.-C. He W.-Z. Liu L. Huang M. (2021). Senescent immune cells release grancalcin to promote skeletal aging. Cell Metab. 33 (10), 1957–1973.e6. 10.1016/j.cmet.2021.08.009 34614408

[B96] Li M. Zhang D. Peng F. Xie J. Zhang X. Qian S. (2022). Zinc-doped ferric oxyhydroxide nano-layer enhances the bactericidal activity and osseointegration of a magnesium alloy through augmenting the formation of neutrophil extracellular traps. Acta Biomater. 152, 575–592. 10.1016/j.actbio.2022.08.066 36070834

[B97] Li Y. Xu X. Wang H. J. Chen Y. C. Chen Y. Chiu J. (2024). Endoplasmic reticulum protein 72 regulates integrin Mac-1 activity to influence neutrophil recruitment. Arterioscler. Thromb. Vasc. Biol. 44 (3), e82–e98. 10.1161/atvbaha.123.319771 38205640

[B98] Liang H. Huang Y. Gao Q. (2021). Role of non-canonical pyroptosis in sepsis and other inflammatory diseases. Zhong Nan Da Xue Xue Bao Yi Xue Ban. 46 (11), 1276–1284. 10.11817/j.issn.1672-7347.2021.210174 34911863 PMC10929856

[B99] Lin A. Loré K. (2017). Granulocytes: new members of the antigen-presenting cell family. Front. Immunol. 8, 1781. 10.3389/fimmu.2017.01781 29321780 PMC5732227

[B100] Liu C. Zhang A. (2020). ROS-Mediated PERK-eIF2α-ATF4 pathway plays an important role in arsenite-induced L-02 cells apoptosis *via* regulating CHOP-DR5 signaling. Environ. Toxicol. 35 (10), 1100–1113. 10.1002/tox.22946 32506763

[B101] Liu X. Miao Y. Liang H. Diao J. Hao L. Shi Z. (2022). 3D-printed bioactive ceramic scaffolds with biomimetic Micro/nano-HAp surfaces mediated cell fate and promoted bone augmentation of the bone-implant interface *in vivo* . Bioact. Mater 12, 120–132. 10.1016/j.bioactmat.2021.10.016 35087968 PMC8777208

[B102] Liu Q. Yu Y. Liu C. Liu Y. Yuan L. Wang Z. (2023). Effect of La3+ and Mg2+ combined system on bioactivity and osteogenesis of bioinspired La-doped magnesium phosphate composites prepared utilizing the precursor method. J. Mater. Res. Technol. 24, 9523–9536. 10.1016/j.jmrt.2023.05.133

[B103] Liu X. Ou X. Zhang T. Li X. Qiao Q. Jia L. (2024). *In situ* neutrophil apoptosis and macrophage efferocytosis mediated by glycyrrhiza protein nanoparticles for acute inflammation therapy. J. Control Release 369, 215–230. 10.1016/j.jconrel.2024.03.029 38508529

[B104] Lokwani R. Josyula A. Ngo T. B. DeStefano S. Fertil D. Faust M. (2024). Pro-regenerative biomaterials recruit immunoregulatory dendritic cells after traumatic injury. Nat. Mater 23 (1), 147–157. 10.1038/s41563-023-01689-9 37872423 PMC13504775

[B105] Luo Y. Liu H. Zhang Y. Liu Y. Liu S. Liu X. (2023). Metal ions: the unfading stars of bone regeneration-from bone metabolism regulation to biomaterial applications. Biomater. Sci. 11 (22), 7268–7295. 10.1039/d3bm01146a 37800407

[B106] Ma S. Xiao Y. Zhang X. Xu Y. Zhu K. Zhang K. (2024). Dietary exposure to polystyrene microplastics exacerbates liver damage in fulminant hepatic failure *via* ROS production and neutrophil extracellular trap formation. Sci. Total Environ. 907, 167403. 10.1016/j.scitotenv.2023.167403 37820799

[B107] Mao C. Xiang Y. Liu X. Cui Z. Yang X. Yeung K. W. K. (2017). Photo-inspired antibacterial activity and wound healing acceleration by hydrogel embedded with ag/Ag@AgCl/ZnO nanostructures. ACS Nano 11 (9), 9010–9021. 10.1021/acsnano.7b03513 28825807

[B108] Martin K. R. Ohayon D. Witko-Sarsat V. (2015). Promoting apoptosis of neutrophils and phagocytosis by macrophages: novel strategies in the resolution of inflammation. Swiss Med. Wkly. 145, w14056. 10.4414/smw.2015.14056 25701669

[B109] McDonald B. Pittman K. Menezes G. B. Hirota S. A. Slaba I. Waterhouse C. C. M. (2010). Intravascular danger signals guide neutrophils to sites of sterile inflammation. Science 330 (6002), 362–366. 10.1126/science.1195491 20947763

[B110] Mereweather L. J. Constantinescu-Bercu A. Crawley J. T. B. Salles-Crawley I. I. (2023). Platelet-neutrophil crosstalk in thrombosis. Int. J. Mol. Sci. 24 (2), 1266. 10.3390/ijms24021266 36674781 PMC9861587

[B111] Minns D. Smith K. J. Alessandrini V. Hardisty G. Melrose L. Jackson-Jones L. (2021). The neutrophil antimicrobial peptide cathelicidin promotes Th17 differentiation. Nat. Commun. 12 (1), 1285. 10.1038/s41467-021-21533-5 33627652 PMC7904761

[B112] Miralda I. Vashishta A. Rogers M. N. Lamont R. J. Uriarte S. M. (2022). The emerging oral pathogen, Filifactor alocis, extends the functional lifespan of human neutrophils. Mol. Microbiol. 117 (6), 1340–1351. 10.1111/mmi.14911 35437843 PMC9233153

[B113] Moonen C. G. J. de Vries T. J. Rijkschroeff P. Poubelle P. E. Nicu E. A. Loos B. G. (2019). The possible role of neutrophils in the induction of osteoclastogenesis. J. Immunol. Res. 2019, 1–14. 10.1155/2019/8672604 31637266 PMC6766092

[B114] Morandini L. Avery D. Angeles B. Winston P. Martin R. K. Donahue H. J. (2023). Reduction of neutrophil extracellular traps accelerates inflammatory resolution and increases bone formation on titanium implants. Acta Biomater. 166, 670–684. 10.1016/j.actbio.2023.05.016 37187302 PMC10330750

[B115] Morgenstern M. Kühl R. Eckardt H. Acklin Y. Stanic B. Garcia M. (2018). Diagnostic challenges and future perspectives in fracture-related infection. Injury 49 (Suppl. 1), S83–S90. 10.1016/s0020-1383(18)30310-3 29929701

[B116] Mukhopadhyay A. Tsukasaki Y. Chan W. C. Le J. P. Kwok M. L. Zhou J. (2024). trans-Endothelial neutrophil migration activates bactericidal function *via* Piezo1 mechanosensing. Immunity 57 (1), 52–67.e10. 10.1016/j.immuni.2023.11.007 38091995 PMC10872880

[B117] Németh T. Sperandio M. Mócsai A. (2020). Neutrophils as emerging therapeutic targets. Nat. Rev. Drug Discov. 19 (4), 253–275. 10.1038/s41573-019-0054-z 31969717

[B118] Ng L. G. Qin J. S. Roediger B. Wang Y. Jain R. Cavanagh L. L. (2011). Visualizing the neutrophil response to sterile tissue injury in mouse dermis reveals a three-phase cascade of events. J. Invest Dermatol 131 (10), 2058–2068. 10.1038/jid.2011.179 21697893

[B119] O'Neill E. Awale G. Daneshmandi L. Umerah O. Lo K. W. H. (2018). The roles of ions on bone regeneration. Drug Discov. Today 23 (4), 879–890. 10.1016/j.drudis.2018.01.049 29407177

[B120] Oakes P. W. Patel D. C. Morin N. A. Zitterbart D. P. Fabry B. Reichner J. S. (2009). Neutrophil morphology and migration are affected by substrate elasticity. Blood 114 (7), 1387–1395. 10.1182/blood-2008-11-191445 19491394 PMC2727411

[B121] Ong G. Logue S. E. (2023). Unfolding the interactions between endoplasmic reticulum stress and oxidative stress. Antioxidants (Basel) 12 (5), 981. 10.3390/antiox12050981 37237847 PMC10215201

[B122] Pattison D. I. Davies M. J. Hawkins C. L. (2012). Reactions and reactivity of myeloperoxidase-derived oxidants: differential biological effects of hypochlorous and hypothiocyanous acids. Free Radic. Res. 46 (8), 975–995. 10.3109/10715762.2012.667566 22348603

[B123] Peng F. Xie J. Liu H. Zheng Y. Qian X. Zhou R. (2023). Shifting focus from bacteria to host neutrophil extracellular traps of biodegradable pure Zn to combat implant centered infection. Bioact. Mater 21, 436–449. 10.1016/j.bioactmat.2022.09.004 36185738 PMC9483647

[B124] Pettygrove B. A. Kratofil R. M. Alhede M. Jensen P. Ø. Newton M. Qvortrup K. (2021). Delayed neutrophil recruitment allows nascent *Staphylococcus aureus* biofilm formation and immune evasion. Biomaterials 275, 120775. 10.1016/j.biomaterials.2021.120775 34243039 PMC8325624

[B125] Ponzetti M. Rucci N. (2019). Updates on osteoimmunology: what's new on the cross-talk between bone and immune system. Front. Endocrinol. (Lausanne) 10, 236. 10.3389/fendo.2019.00236 31057482 PMC6482259

[B126] Porter L. M. Cowburn A. S. Farahi N. Deighton J. Farrow S. N. Fiddler C. A. (2017). Hypoxia causes IL-8 secretion, charcot leyden crystal formation, and suppression of corticosteroid-induced apoptosis in human eosinophils. Clin. Exp. Allergy 47 (6), 770–784. 10.1111/cea.12877 28000962

[B127] Prame Kumar K. Nicholls A. J. Wong C. H. Y. (2018). Partners in crime: neutrophils and monocytes/macrophages in inflammation and disease. Cell Tissue Res. 371 (3), 551–565. 10.1007/s00441-017-2753-2 29387942 PMC5820413

[B128] Puga I. Cols M. Barra C. M. He B. Cassis L. Gentile M. (2011). B cell-helper neutrophils stimulate the diversification and production of immunoglobulin in the marginal zone of the spleen. Nat. Immunol. 13 (2), 170–180. 10.1038/ni.2194 22197976 PMC3262910

[B129] Qian G. Zhang L. Wang G. Zhao Z. Peng S. Shuai C. (2021a). 3D printed Zn-doped mesoporous silica-incorporated Poly-L-lactic acid scaffolds for bone repair. Int. J. Bioprint 7 (2), 346. 10.18063/ijb.v7i2.346 33997435 PMC8114096

[B130] Qian G. Zhang L. Liu X. Wu S. Peng S. Shuai C. (2021b). Silver-doped bioglass modified scaffolds: a sustained antibacterial efficacy. Mater. Sci. Eng. C 129, 112425. 10.1016/j.msec.2021.112425 34579875

[B131] Rabel K. Kohal R.-J. Steinberg T. Tomakidi P. Rolauffs B. Adolfsson E. (2020). Controlling osteoblast morphology and proliferation *via* surface micro-topographies of implant biomaterials. Sci. Rep. 10 (1), 12810. 10.1038/s41598-020-69685-6 32732908 PMC7393177

[B132] Rani Talla U. Bozonet S. Parker H. Hampton M. Vissers M. (2016). Inhibition of neutrophil apoptosis and initiation of an autophagy-like process in hypoxia and effects on neutrophil function. Free Radic. Biol. Med. 100, S63. 10.1016/j.freeradbiomed.2016.10.166

[B133] Saberi A. Kouhjani M. Mohammadi M. Hosta-Rigau L. (2023). Novel scaffold platforms for simultaneous induction osteogenesis and angiogenesis in bone tissue engineering: a cutting-edge approach. J. Nanobiotechnology 21 (1), 351. 10.1186/s12951-023-02115-7 37770928 PMC10536787

[B134] Saffarzadeh M. Juenemann C. Queisser M. A. Lochnit G. Barreto G. Galuska S. P. (2012). Neutrophil extracellular traps directly induce epithelial and endothelial cell death: a predominant role of histones. PLoS One 7 (2), e32366. 10.1371/journal.pone.0032366 22389696 PMC3289648

[B135] Sarkar A. Hellberg L. Bhattacharyya A. Behnen M. Wang K. Lord J. M. (2012). Infection with Anaplasma phagocytophilum activates the phosphatidylinositol 3-Kinase/Akt and NF-κB survival pathways in neutrophil granulocytes. Infect. Immun. 80 (4), 1615–1623. 10.1128/iai.05219-11 22252875 PMC3318406

[B136] Sarkar A. Möller S. Bhattacharyya A. Behnen M. Rupp J. van Zandbergen G. (2015). Mechanisms of apoptosis inhibition in chlamydia pneumoniae-infected neutrophils. Int. J. Med. Microbiol. 305 (6), 493–500. 10.1016/j.ijmm.2015.04.006 26005182

[B137] Scapini P. Cassatella M. A. (2014). Social networking of human neutrophils within the immune system. Blood 124 (5), 710–719. 10.1182/blood-2014-03-453217 24923297

[B138] Scherlinger M. Richez C. Tsokos G. C. Boilard E. Blanco P. (2023). The role of platelets in immune-mediated inflammatory diseases. Nat. Rev. Immunol. 23 (8), 495–510. 10.1038/s41577-023-00834-4 36707719 PMC9882748

[B139] Schmidt-Bleek K. Schell H. Schulz N. Hoff P. Perka C. Buttgereit F. (2012). Inflammatory phase of bone healing initiates the regenerative healing Cascade. Cell Tissue Res. 347 (3), 567–573. 10.1007/s00441-011-1205-7 21789579

[B140] Schneider A. H. Taira T. M. Públio G. A. da Silva Prado D. Donate Yabuta P. B. Dos Santos J. C. (2024). Neutrophil extracellular traps mediate bone erosion in rheumatoid arthritis by enhancing RANKL-induced osteoclastogenesis. Br. J. Pharmacol. 181 (3), 429–446. 10.1111/bph.16227 37625900

[B141] Schuster S. Hurrell B. Tacchini-Cottier F. (2013). Crosstalk between neutrophils and dendritic cells: a context-dependent process. J. Leukoc. Biol. 94 (4), 671–675. 10.1189/jlb.1012540 23250891

[B142] Shi W. Feng Y. Tang J. Xu Y. Wang W. Zhang L. (2024). A genetically engineered “Reinforced Concrete” scaffold regulates the N2 neutrophil Innate immune Cascade to repair bone defects. Adv. Healthc. Mater 13 (18), e2304585. 10.1002/adhm.202304585 38411324

[B143] Shu L.-Z. Zhang X.-L. Ding Y.-D. Lin H. (2024). From inflammation to bone formation: the intricate role of neutrophils in skeletal muscle injury and traumatic heterotopic ossification. Exp. Mol. Med. 56 (7), 1523–1530. 10.1038/s12276-024-01270-7 38945957 PMC11297321

[B144] Singh P. Hu P. Hoggatt J. Moh A. Pelus L. M. (2012). Expansion of bone marrow neutrophils following G-CSF administration in mice results in osteolineage cell apoptosis and mobilization of hematopoietic stem and progenitor cells. Leukemia 26 (11), 2375–2383. 10.1038/leu.2012.117 22543963 PMC3410045

[B145] Soehnlein O. Zernecke A. Eriksson E. E. Rothfuchs A. G. Pham C. T. Herwald H. (2008). Neutrophil secretion products pave the way for inflammatory monocytes. Blood 112 (4), 1461–1471. 10.1182/blood-2008-02-139634 18490516 PMC3400540

[B146] Spiegel D. A. Shrestha O. P. Rajbhandary T. Bijukachhe B. Sitoula P. Banskota B. (2010). Epidemiology of surgical admissions to a children's disability hospital in Nepal. World J. Surg. 34 (5), 954–962. 10.1007/s00268-010-0487-3 20177682

[B147] Stroka K. M. Aranda-Espinoza H. (2011). Endothelial cell substrate stiffness influences neutrophil transmigration *via* myosin light chain kinase-dependent cell contraction. Blood 118 (6), 1632–1640. 10.1182/blood-2010-11-321125 21652678 PMC3156049

[B148] Subramanian Vignesh K. Deepe G. S. (2016). Immunological orchestration of zinc homeostasis: the battle between host mechanisms and pathogen defenses. Arch. Biochem. Biophys. 611, 66–78. 10.1016/j.abb.2016.02.020 26921502 PMC4996772

[B149] Suliman S. Mieszkowska A. Folkert J. Rana N. Mohamed-Ahmed S. Fuoco T. (2022). Immune-instructive copolymer scaffolds using plant-derived nanoparticles to promote bone regeneration. Inflamm. Regen. 42 (1), 12. 10.1186/s41232-022-00196-9 35366945 PMC8977008

[B150] Takegahara N. Kim H. Choi Y. (2024). Unraveling the intricacies of osteoclast differentiation and maturation: insight into novel therapeutic strategies for bone-destructive diseases. Exp. Mol. Med. 56 (2), 264–272. 10.1038/s12276-024-01157-7 38297158 PMC10907717

[B151] Tan H. Zhang S. Zhang Z. Zhang J. Wang Z. Liao J. (2024). Neutrophil extracellular traps promote M1 macrophage polarization in gouty inflammation *via* targeting hexokinase-2. Free Radic. Biol. Med. 224, 540–553. 10.1016/j.freeradbiomed.2024.09.009 39277122

[B152] Tao H. Wang Q. Chen K. Zhu P. Gu Y. Geng D. (2024). Metal ion metabolism and osteoporosis: possible implications for pharmaceutical biotechnology and tissue engineering. Sci. China Life Sci. 67 (8), 1763–1765. 10.1007/s11427-023-2541-x 38676813

[B153] Telang S. (2018). Lactoferrin: a critical player in neonatal host defense. Nutrients 10 (9), 1228. 10.3390/nu10091228 30181493 PMC6165050

[B154] Teng T.-S. Ji A.-L. Ji X.-Y. Li Y.-Z. (2017). Neutrophils and immunity: from bactericidal action to being conquered. J. Immunol. Res. 2017, 1–14. 10.1155/2017/9671604 28299345 PMC5337389

[B155] Thakur M. Junho C. V. C. Bernhard S. M. Schindewolf M. Noels H. Döring Y. (2023). NETs-Induced thrombosis impacts on cardiovascular and chronic kidney disease. Circ. Res. 132 (8), 933–949. 10.1161/circresaha.123.321750 37053273 PMC10377271

[B156] Thompson A. A. R. Elks P. M. Marriott H. M. Eamsamarng S. Higgins K. R. Lewis A. (2014). Hypoxia-inducible factor 2α regulates key neutrophil functions in humans, mice, and zebrafish. Blood 123 (3), 366–376. 10.1182/blood-2013-05-500207 24196071 PMC3894493

[B157] Toller-Kawahisa J. E. Hiroki C. H. Silva C. M. d.S. Nascimento D. C. Públio G. A. Martins T. V. (2023). The metabolic function of pyruvate kinase M2 regulates reactive oxygen species production and microbial killing by neutrophils. Nat. Commun. 14 (1), 4280. 10.1038/s41467-023-40021-6 37460614 PMC10352279

[B158] Vatansever F. de Melo W. C. M. A. Avci P. Vecchio D. Sadasivam M. Gupta A. (2013). Antimicrobial strategies centered around reactive oxygen species--bactericidal antibiotics, photodynamic therapy, and beyond. FEMS Microbiol. Rev. 37 (6), 955–989. 10.1111/1574-6976.12026 23802986 PMC3791156

[B159] Walmsley S. R. Print C. Farahi N. Peyssonnaux C. Johnson R. S. Cramer T. (2005). Hypoxia-induced neutrophil survival is mediated by HIF-1α–dependent NF-κB activity. J. Exp. Med. 201 (1), 105–115. 10.1084/jem.20040624 15630139 PMC2212759

[B160] Wang P.-Y. Li W.-T. Yu J. Tsai W.-B. (2012). Modulation of osteogenic, adipogenic and myogenic differentiation of mesenchymal stem cells by submicron grooved topography. J. Mater Sci. Mater Med. 23 (12), 3015–3028. 10.1007/s10856-012-4748-6 22903603

[B161] Wang C. Zhang R. Wei X. Lv M. Jiang Z. (2020). Metalloimmunology: the metal ion-controlled immunity. Adv. Immunol. 145, 187–241. 10.1016/bs.ai.2019.11.007 32081198

[B162] Wang J.-F. Wang Y.-P. Xie J. Zhao Z.-Z. Gupta S. Guo Y. (2021). Upregulated PD-L1 delays human neutrophil apoptosis and promotes lung injury in an experimental mouse model of sepsis. Blood 138 (9), 806–810. 10.1182/blood.2020009417 34473230

[B163] Wang H. Kim S. J. Lei Y. Wang S. Wang H. Huang H. (2024a). Neutrophil extracellular traps in homeostasis and disease. Signal Transduct. Target Ther. 9 (1), 235. 10.1038/s41392-024-01933-x 39300084 PMC11415080

[B164] Wang L. Zhang G. Gao Y. Dai T. Yu J. Liu Y. (2024b). Extracellular vesicles derived from neutrophils accelerate bone regeneration by promoting osteogenic differentiation of BMSCs. ACS Biomater. Sci. Eng. 10 (6), 3868–3882. 10.1021/acsbiomaterials.4c00106 38703236 PMC11167592

[B165] Wang Z. Chu Y. Du J. Hu Y. Wang H. Liu H. (2025a). Accelerating repair of infected bone defects through post-reinforced injectable hydrogel mediated antibacterial/immunoregulatory microenvironment at bone-hydrogel interface. Carbohydr. Polym. 351, 123082. 10.1016/j.carbpol.2024.123082 39779005

[B166] Wang N. Tao Y. Yang Y. Jin Y. Zhang H. Li C. (2025b). Disrupting the activity of endogenous gas neurotransmitters: a therapeutic strategy using engineered metal-organic frameworks for cancer. Med. Gas. Res. 15 (1), 142–144. 10.4103/mgr.medgasres-d-24-00046 39436187 PMC11515053

[B167] Wei Y. Wang J. Wu S. Zhou R. Zhang K. Zhang Z. (2022). Nanomaterial-based zinc ion interference therapy to combat bacterial infections. Front. Immunol. 13, 899992. 10.3389/fimmu.2022.899992 35844505 PMC9279624

[B168] Wei P. Zhou J. Xiong S. Yi F. Xu K. Liu M. (2024). Chestnut-inspired hollow hydroxyapatite 3D printing scaffolds accelerate bone regeneration by recruiting calcium ions and regulating inflammation. ACS Appl. Mater Interfaces 16 (8), 9768–9786. 10.1021/acsami.3c17087 38349802

[B169] Won J.-E. Lee Y. S. Park J.-H. Lee J.-H. Shin Y. S. Kim C.-H. (2020). Hierarchical microchanneled scaffolds modulate multiple tissue-regenerative processes of immune-responses, angiogenesis, and stem cell homing. Biomaterials 227, 119548. 10.1016/j.biomaterials.2019.119548 31670033

[B170] Wu D. Li J. Xu S. Xie Q. Pan Y. Liu X. (2020). Engineering Fe-N doped graphene to mimic biological functions of NADPH oxidase in cells. J. Am. Chem. Soc. 142 (46), 19602–19610. 10.1021/jacs.0c08360 33108194

[B171] Xing Z. Lu C. Hu D. Miclau T. Marcucio R. S. (2010). Rejuvenation of the inflammatory system stimulates fracture repair in aged mice. J. Orthop. Res. 28 (8), 1000–1006. 10.1002/jor.21087 20108320 PMC2892015

[B172] Xiong S. Zhang Y. Zeng J. Zhou J. Liu S. Wei P. (2024). DLP fabrication of HA scaffold with customized porous structures to regulate immune microenvironment and macrophage polarization for enhancing bone regeneration. Mater Today Bio 24, 100929. 10.1016/j.mtbio.2023.100929 38229884 PMC10789648

[B173] Xu X. Chen Z. Xiao L. Xu Y. Xiao N. Jin W. (2023). Nanosilicate-functionalized nanofibrous membrane facilitated periodontal regeneration potential by harnessing periodontal ligament cell-mediated osteogenesis and immunomodulation. J. Nanobiotechnology 21 (1), 223. 10.1186/s12951-023-01982-4 37443072 PMC10339597

[B174] Xu Y. Wang D. Zhou S. Yu D. Deng A. Lu X. (2025). Apoptotic bodies from neutrophil-like cells *in situ* regulating macrophage polarization for autoimmune disease treatment. Nano Res. 18 (9), 94907775. 10.26599/nr.2025.94907775

[B175] Yamasawa H. Oshikawa K. Ohno S. Sugiyama Y. (2004). Macrolides inhibit epithelial cell-mediated neutrophil survival by modulating granulocyte macrophage colony-stimulating factor release. Am. J. Respir. Cell Mol. Biol. 30 (4), 569–575. 10.1165/rcmb.2003-0105oc 14551160

[B176] Yang P. Luo X. Li J. Zhang T. Gao X. Hua J. (2021). Ionizing radiation upregulates glutamine metabolism and induces cell death *via* accumulation of reactive oxygen species. Oxid. Med. Cell Longev. 2021, 5826932. 10.1155/2021/5826932 35028001 PMC8749225

[B177] Ye Q. Harmsen M. C. van Luyn M. J. A. Bank R. A. (2010). The relationship between collagen scaffold cross-linking agents and neutrophils in the foreign body reaction. Biomaterials 31 (35), 9192–9201. 10.1016/j.biomaterials.2010.08.049 20828809

[B178] Yu Y.-M. Lu Y.-P. Zhang T. Zheng Y.-F. Liu Y.-S. Xia D.-D. (2024). Biomaterials science and surface engineering strategies for dental peri-implantitis management. Mil. Med. Res. 11 (1), 29. 10.1186/s40779-024-00532-9 38741175 PMC11089802

[B179] Zeng J. Xiong S. Zhou J. Wei P. Guo K. Wang F. (2023). Hollow hydroxyapatite microspheres loaded with rhCXCL13 to recruit BMSC for osteogenesis and synergetic angiogenesis to promote bone regeneration in bone defects. Int. J. Nanomedicine 18, 3509–3534. 10.2147/ijn.s408905 37404852 PMC10317543

[B180] Zhang X. Wang X. Wu T. Li B. Liu T. Wang R. (2015). Isoliensinine induces apoptosis in triple-negative human breast cancer cells through ROS generation and p38 MAPK/JNK activation. Sci. Rep. 5, 12579. 10.1038/srep12579 26219228 PMC4518223

[B181] Zhao R.-Z. Jiang S. Zhang L. Yu Z.-B. (2019). Mitochondrial electron transport chain, ROS generation and uncoupling. Int. J. Mol. Med. 44 (1). 10.3892/ijmm.2019.4188 31115493 PMC6559295

[B182] Zheng W. Li J. Li J. Bie N. Wei Z. Qin J. (2024). *In-situ* nanoplatform with synergistic neutrophil intervention and chemotherapy to prevent postoperative tumor recurrence and metastasis. J. Control Release 375, 316–330. 10.1016/j.jconrel.2024.09.011 39251139

[B183] Zhou C.-X. Li L. Ma Y.-G. Li B.-N. Li G. Zhou Z. (2018). A bioactive implant *in situ* and long-term releases combined drugs for treatment of osteoarticular tuberculosis. Biomaterials 176, 50–59. 10.1016/j.biomaterials.2018.05.039 29857274

[B184] Zhou J. Xiong S. Liu M. Yang H. Wei P. Yi F. (2023). Study on the influence of scaffold morphology and structure on osteogenic performance. Front. Bioeng. Biotechnol. 11, 1127162. 10.3389/fbioe.2023.1127162 37051275 PMC10083331

[B185] Zhu S. Bennett S. Kuek V. Xiang C. Xu H. Rosen V. (2020). Endothelial cells produce angiocrine factors to regulate bone and cartilage *via* versatile mechanisms. Theranostics 10 (13), 5957–5965. 10.7150/thno.45422 32483430 PMC7255007

[B186] Zhu Y. P. Speir M. Tan Z. Lee J. C. Nowell C. J. Chen A. A. (2023). NET formation is a default epigenetic program controlled by PAD4 in apoptotic neutrophils. Sci. Adv. 9 (51), eadj1397. 10.1126/sciadv.adj1397 38117877 PMC10732518

[B187] Zhu J. Xie R. Gao R. Zhao Y. Yodsanit N. Zhu M. (2024). Multimodal nanoimmunotherapy engages neutrophils to eliminate *Staphylococcus aureus* infections. Nat. Nanotechnol. 19 (7), 1032–1043. 10.1038/s41565-024-01648-8 38632494 PMC12126137

